# Valproic acid potentiates the anticancer activity of capecitabine *in vitro* and *in vivo* in breast cancer models via induction of thymidine phosphorylase expression

**DOI:** 10.18632/oncotarget.6802

**Published:** 2015-12-31

**Authors:** Manuela Terranova-Barberio, Maria Serena Roca, Andrea Ilaria Zotti, Alessandra Leone, Francesca Bruzzese, Carlo Vitagliano, Giosuè Scogliamiglio, Domenico Russo, Giovanni D'Angelo, Renato Franco, Alfredo Budillon, Elena Di Gennaro

**Affiliations:** ^1^ Experimental Pharmacology Unit, Istituto Nazionale Tumori Fondazione G. Pascale - IRCCS, Naples, Italy; ^2^ Pathology Unit, Istituto Nazionale Tumori Fondazione G. Pascale - IRCCS, Naples, Italy; ^3^ Institute of Protein Biochemistry, National Research Council, Naples, Italy

**Keywords:** HDAC inhibitor, valproic acid, thymidine phosphorylase, breast cancer, capecitabine

## Abstract

The prognosis of patients with metastatic breast cancer remains poor, and thus novel therapeutic approaches are needed. Capecitabine, which is commonly used for metastatic breast cancer in different settings, is an inactive prodrug that takes advantage of elevated levels of thymidine phosphorylase (TP), a key enzyme that is required for its conversion to 5-fluororacil, in tumors. We demonstrated that histone deacetylase inhibitors (HDACi), including low anticonvulsant dosage of VPA, induced the dose- and time-dependent up-regulation of TP transcript and protein expression in breast cancer cells, but not in the non-tumorigenic breast MCF-10A cell line. Through the use of siRNA or isoform-specific HDACi, we demonstrated that HDAC3 is the main isoform whose inhibition is involved in the modulation of TP. The combined treatment with capecitabine and HDACi, including valproic acid (VPA), resulted in synergistic/additive antiproliferative and pro-apoptotic effects in breast cancer cells but not in TP-knockout cells, both *in vitro* and *in vivo*, highlighting the crucial role of TP in the synergism observed. Overall, this study suggests that the combination of HDACi (e.g., VPA) and capecitabine is an innovative antitumor strategy that warrants further clinical evaluation for the treatment of metastatic breast cancer.

## INTRODUCTION

Breast cancer remains one of the most serious and common diseases and is second only to lung cancer as the leading cause of cancer death in women [1]. Although advances in breast cancer treatment have led to the development of novel therapeutics in the last years, metastatic breast cancer is largely an incurable disease.

As a monotherapy, capecitabine, an oral prodrug of 5-fluorourcacil (5-FU), is one of the mainstays for the treatment of patients with metastatic breast cancer who are ineligible for or who are pretreated with a more intensive anthracycline- and/or taxane-based regimen. Moreover, capecitabine is approved for use in combination with docetaxel for the treatment of breast cancer patients who have received prior therapy with anthracycline, taxane, and trastuzumab. Furthermore, due to its tolerability and efficacy as a single agent and its lack of cross-resistance with other chemotherapeutics, capecitabine may also be considered a preferred partner to be assessed in novel combination regimens [2–4]. After its absorption as an intact molecule, capecitabine is converted into 5-FU in the gastrointestinal tract through a three-step process. The key step, the conversion of 5′-deoxy-5-fluorouridine (5′-DFUR) into active 5-FU, occurs primarily in the tumor and is catalyzed by thymidine phosphorylase (TP). Subsequently, 5-FU is metabolized into two active cytotoxic metabolites. The first, 5-fluoro-2-deoxyuridine monophosphate (FdUMP), interferes with DNA synthesis through a reduction in thymidine production following the inhibition of thymidylate synthase (TS). The second, 5-fluorouridine triphosphate (FUTP), inhibits RNA and protein synthesis by competing with uridine-triphosphate for incorporation into the RNA strand [2, 5]. Therefore, TP represents the rate-limiting enzyme in the activation of 5′-DFUR and capecitabine, and its elevated level in many tumors allows for high local concentrations of the active drug [2]. Indeed, in breast cancer, TP has been considered a predictive marker for clinical response to capecitabine [6, 7], which suggests that an increase in TP expression might enhance sensitivity of tumor cells to this prodrug. Conversely, the overexpression of TS has been associated with aggressive breast cancer phenotypes and a worse prognosis, particularly in patients with breast cancer who are treated with 5-FU-based chemotherapy [8].

Through the regulation of acetylation of histone and non-histone proteins, histone deacetylases (HDACs) control cellular functions such as the cell cycle, proliferation, survival, DNA repair and differentiation. Their expression is frequently altered in hematologic and solid tumors [9]. Class-I HDACs (1–3, 8) are predominantly expressed in the nucleus and are the major mediators of histone deacetylation, whereas class-IIa HDACs (4, 5, 7, 9) and class-IIb HDACs (6 and 10) either shuttle between the nucleus and the cytoplasm or are predominantly expressed in the cytoplasm where they function to deacetylate non-histone proteins [9]. A large number of histone deacetylase inhibitors (HDACi) are currently in clinical development as anticancer agents, and three (vorinostat, romidepsin and belinostat^1^) have been approved by the FDA for the treatment of cutaneous T-cell lymphoma [10–12]. Moreover, panobinostat was the first HDACi approved as a combination therapy to treat recurrent multiple myeloma [13]^2^. In solid tumors including breast cancer, HDACi have failed to show considerable antitumor activity as single agents and are more active in combination with radiotherapy, chemotherapy or other biologicals [14]. Recently, based on the results of a phase-II randomized trial, the HDACi entinostat has been designated as a breakthrough therapy for the treatment of recurrent/metastatic estrogen receptor-positive breast cancer when given in combination with exemestane in postmenopausal women who progressed following non-steroidal aromatase inhibitor therapy [15].

Our group demonstrated the synergistic antitumor activity of HDACi in combination with several anticancer agents, including 5-FU and capecitabine [16–19]. In details, we have previously demonstrated, that the HDACi vorinostat in combination with capecitabine produces a synergistic antitumor effects by up-regulating, *in vitro* and *in vivo*, in colorectal cancer cells but not in *ex vivo* treated peripheral blood lymphocytes, the mRNA and protein expression of TP [18].

Valproic acid (VPA) is a generic low-cost anticonvulsant and mood stabilizer that has been used for over 40 years and that demonstrates HDAC inhibitory activity and anticancer properties [20]. Compared with other HDACi, VPA has a good safety profile, and neovestibular symptoms, fatigue and somnolence are the only dose-limiting toxicities [20]. Several phase-I and phase-II studies of VPA in hematologic and solid malignancies, including breast cancer, showed that VPA, either as a monotherapy or in combination with other agents, was reasonably well tolerated and resulted in some encouraging responses [20–24].

In the present study, we demonstrated that several HDACi including VPA down-regulate TS and up-regulate TP mRNA and protein expression in breast cancer cell lines. VPA modulates TP at the transcriptional level, and this alteration primarily involves the HDAC3 isoform. We showed that these effects were obtained even with low doses of VPA easily achieved in epileptic patients treated with common anticonvulsant dosage. Moreover, through the use of a stable TP-knockout cell model, we demonstrated that TP has a critical role both *in vitro* and *in vivo* in the synergistic antitumor effects of VPA in combination with 5′-DFUR and capecitabine, respectively.

## RESULTS

### Cytotoxic effects of HDACi and 5′-DFUR in breast cancer cells

Multiple cell lines that represent the molecular diversity of breast cancer were equally sensitive to the cytotoxic effect of different HDACi (VPA, vorinostat, entinostat and panobinostat) independently of subtype, p53, RAS, hormone receptor expression (ER and PR), HER2 status (Table 1) or the basal expression of HDAC isoforms, TS or TP proteins (Supplementary Figure S1). Conversely, in the two triple negative MDA-MB231 and MDA-MB468 cell lines, lower mRNA and protein expression of TP correlated with lower sensitivity to the capecitabine metabolite 5′-DFUR (Table 1 and Supplementary Figure S1A and S1B), which confirms the critical role of TP expression for fluoropyrimidine-induced cytotoxicity.

**Table 1:** Characteristics and sensitivity of breast cancer cell lines to vorinostat, panobinostat, valproic acid, entinostat and 5′-DFUR

cell lines	breast cancer subtype	p53 status	RAS status	additional features	IC_50_ vor, μM (± SD)	IC_50_ pan, μM (± SD)	IC_50_ VPA, mM (± SD)	IC_50_ ent, μM (± SD)	IC_50_ 5′-DFUR, μM (± SD)
MCF-7	Luminal B	wt	wt	ER^+^/PR^+^ /Her2^−^	0.77 (± 0.28)	0.029 (± 0.016)	2.22 (± 0.74)	0.46 (± 0.023)	2.03 (± 0.18)
SKBR3	Basal like	mut	wt	ER^−^/PR^−^/Her2^+++^	1.24 (± 0.22)	0.046 (± 0.0066)	1.69 (± 0.54)	0.83 (± 0.3)	1.99 (± 0.57)
MDA-MB231	Triple negative Basal like	mut	mut	ER^−^/PR^−^/Her2^−^	0.92 (± 0.17)	0.10 (± 0.028)	1.60 (± 0.17)	0.2 (± 0.035)	10.4 (± 3.78)
MDA-MB468	Triple negative Basal like	mut	wt	ER^−^/PR^−^/Her2^−^	0.76 (± 0.12)	0.11 (± 0.011)	3.19 (± 0.75)	1.01 (± 0.21)	12.14 (± 1.09)

IC_50_ values computed at 96 hours of treatment (mean ± SD from at least three separate experiments performed in quadruplicates).

*Abbreviations:* vor (vorinostat); pan (panobinostat), VPA (valproic acid); ent (entinostat); 5′-DFUR (5′-deoxy-5-fluorouridine); mut (mutant); wt (wild-type); ER (estrogen receptor); PR (progesterone receptor); Her2 (human epidermal growth factor receptor 2); SD (standard deviation).

MDA-MB231, together with the non-tumorigenic cell line MCF-10A, is the only breast cancer cell line to express the mesenchymal marker vimentin, while all the other three breast cancer cell lines (MCF-7, SKBR3 and MDA-MB468) express the epithelial marker E-cadherin (Supplementary Figure S1A). Moreover MDA-MB231 and MCF-10A showed a similar high basal expression of HDAC3 and HDAC6 enzymes compared to the other tumorigenic cell lines analysed (Supplementary Figure S1C).

### HDACi modulate TS and TP proteins

**Figure 1:**
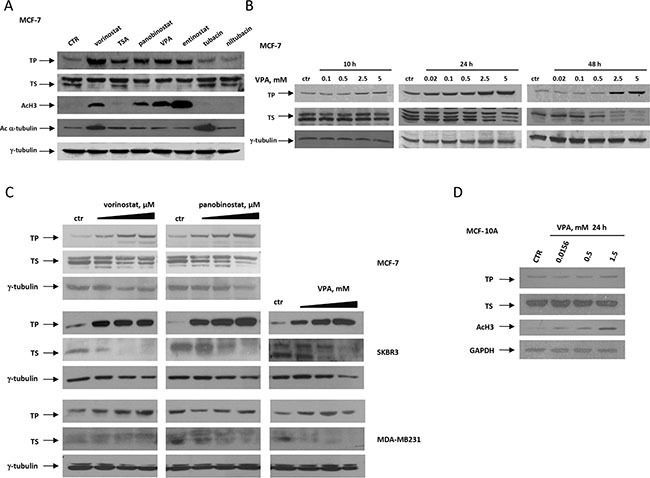
HDACi modulates TP and TS expression in breast cancer cell lines in a dose- and time-dependent manner Protein expression was determined by western blot. **A.** TP, TS, acetyl-H3 and acetyl-α-tubulin proteins evaluated in MCF-7 cells untreated or treated for 24 hours with different HDACi at concentration corresponding to IC_50_^72h^. **B.** TP and TS proteins evaluated in MCF-7 untreated or treated with increased doses of VPA for 10, 24 or 48 hours. **C.** TP and TS proteins evaluated in MCF-7, SKBR3 and MDA-MB231 breast cancer cells untreated or treated with increased doses of vorinostat, panobinostat or VPA for 48 hours. **D.** TP, TS and acetyl-H3 proteins evaluated in non-tumorigenic MCF-10A cells untreated or treated with increased doses of VPA for 24 hours. For A, B, C and D, γ-tubulin or GAPDH were used as protein loading control.

We then demonstrated that HDACi with different specificity are able to modulate TP and TS protein levels in all breast cancer cell lines, as previously described by us and other groups in different tumor models [18, 25, 26]. Both pan-HDACi (e.g., vorinostat, trichostatin-A, panobinostat) and class-I/IIa (e.g., VPA) or class-I HDACi (e.g., entinostat) are able to up-regulate TP and down-regulate TS expression in MCF-7 cells. However, the HDAC6-selective inhibitor tubacin is unable to modulate TS protein and only slightly induces TP expression compared with its inactive homologue niltubacin [27] (Figure 1A). Time- and dose-dependent modulation of TP and TS proteins by vorinostat, panobinostat and VPA were confirmed in all cell lines within 10 hours of treatment. This modulation was independent of basal expression of both enzymes (Figure 1B, 1C and Supplementary Figure S2). Notably, even low doses of HDACi are able to up-regulate the level of TP and down-regulate the level of TS (Figure 1B and 1C). A time-course VPA removal experiment revealed that the VPA-induced up-regulation of TP protein lasts as long as 24 hours after treatment, while the down-regulation of TS is prolonged up to 48 hours after washout (Supplementary Figure S3).

Notably, in the non-tumorigenic cell line MCF-10A, although increased histone-H3 acetylation was evident after 24 hours, we did not observe TP induction or TS down-regulation by VPA (Figure 1D). Moreover a time-course experiment showed no major changes of both proteins up to 72 h of VPA treatment (Supplementary Figure S2B), confirming that the modulation of both enzymes is confined to cancer cells.

### HDACi induce TP expression at the transcriptional level

We next evaluated the mechanism of HDACi-mediated induction of TP protein that is observed in breast cancer cells.

**Figure 2:**
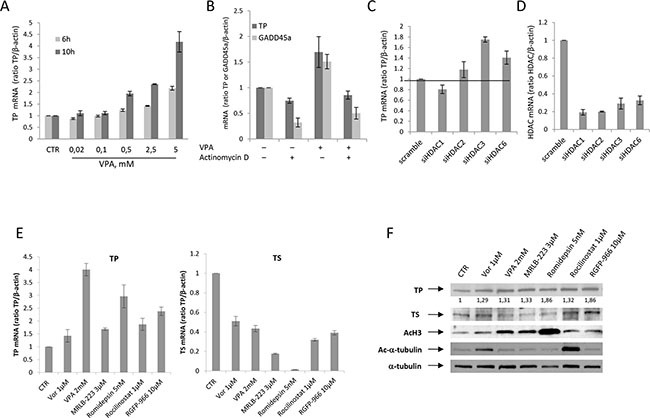
HDACi induced TP expression at the transcriptional level **A.** TP mRNA expression was evaluated by qRealTime-PCR in untreated MCF-7 cells and MCF-7 cells that were treated with increased doses of VPA for 6 or 10 hours. **B.** MCF-7 cells that were untreated or treated with VPA (2.5 mM) and/or actinomycin D (1μg/mL) for 10 hours. The expression levels of TP and GADD45a mRNA were determined by qRealTime-PCR. **C.** TP mRNA expression was determined by qRealTime-PCR in MCF-7 cells that were exposed to HDAC1, HDAC2, HDAC3, HDAC6 or scrambled (33 nM) siRNA for 24 hours. **D.** The efficiency of HDAC1, HDAC2, HDAC3 and HDAC6 knockdown was verified by qRealTime-PCR. **E.** The expression levels of TP and TS mRNA were evaluated by qRealTime-PCR in untreated MCF-7 cells and in MCF-7 cells that were untreated or treated with selective HDACi at the indicated concentrations for 24 hours. For A, B, C, D and E, β-actin was used as the housekeeping control gene to which the expression levels obtained by qRealTime-PCR were normalized. **F.** The expression of TP, TS, acetylated H3 and acetylated α-tubulin proteins was determined by western blot in untreated cells and in MCF-7 cells that were treated for 48 hours with different HDACi at the indicated concentrations. α-tubulin was used as the protein loading control.

As shown in Figure 2A, the up-regulation of TP protein correlates with a dose- and time-dependent mRNA increase induced by VPA in MCF-7 cells. Moreover, the VPA-mediated increase of TP mRNA within 10 hours was completely blocked by concurrent treatment with the transcriptional inhibitor actinomycin-D. This result is consistent with a transcriptional effect (Figure 2B). GADD45a, a well-known gene that is up-regulated by HDACi at the transcriptional level [28], was evaluated as positive control.

On the basis of the data shown in Figure 1 and to identify if a specific HDAC isoform is involved in the modulation of TP, we treated MCF-7 cells with HDAC1, HDAC2, HDAC3, HDAC6 or scrambled siRNAs (33 nM) for 24 hours. As shown in Figure 2C, HDAC3 silencing produced the best induction of TP transcript, whereas no effect or just a slight increase was observed with HDAC1 or HDAC2 silencing, respectively. An evident but smaller increase was observed with HDAC6 silencing. Moreover, to confirm these data, we evaluated TP transcript in MCF-7 cells that were treated with vorinostat, which may inhibit all four class-I HDACs (1,2,3,8) and HDAC6 [29]. The cells were also treated with VPA, a class-I HDAC inhibitor that may also partially inhibit class-IIa HDACs (4, 5, 7 but not HDAC9) [20]. TP transcript was also evaluated in cells that were treated with the selective-HDAC1-2 inhibitor MRLB-223 [30], the class-I-selective HDACi romidepsin, the selective-HDAC6 inhibitor rocilinostat [31], and the selective-HDAC3 inhibitor RGFP-966 [31]. As shown in Figure 2E, the best induction of TP transcript was observed in cells that were treated with VPA, romidepsin or RGFP-966, which confirms that class-I HDACs, and HDAC3 in particular, are the primary HDACs that are involved in the regulation of TP expression in MCF-7 cells. However, rocilinostat also induced TP expression, albeit to a lesser extent, which suggests that HDAC6 may also be involved. Conversely, TS down-regulation was significantly induced by all HDACi tested but primarily by MRLB-223 and romidepsin, which suggests that HDACs 1 and 2 are probably involved in the modulation of TS. Notably, these data were also confirmed at the protein level, with romidepsin and RGFP-966 as the best inducers of TP and MRLB-223 and romidepsin as the best down-regulators of TS. Because deacetylation of α-tubulin is mediated by HDAC6, we used α-tubulin acetylation as a marker of HDAC6 inhibitor activity, while histone-H3 acetylation was used as a surrogate marker of class-I HDACi activity (Figure 2F). Interestingly, although only vorinostat and rocilinostat were confirmed as HDAC6 inhibitors, being able to induce α-tubulin acetylation, the ability of rocilinostat also to induced histone-H3 acetylation, suggest that the induction of TP expression by this latter agent could not be related to HDAC6 inhibition. We should also note that the comparable dosage of the inhibitors used in these assays was derived from published data and could have influenced the extent of the observed effects [30, 32, 33]. Similarly, HDAC isoform-specific silencing resulted in compensatory modulation of different HDACs (data not shown) that might have affected the modulation of TP expression.

The observed modulation of TS and TP expression prompted us to investigate if, consequently, HDACi (particularly VPA) might increase the sensitivity of breast cancer cells to fluoropyrimidines such as 5′-DFUR and capecitabine.

### *In vitro* synergistic antitumor effects of HDACi in combination with 5′-DFUR in breast cancer cell lines

We first evaluated the cytotoxic effects of different HDACi in combination with 5′-DFUR by performing a median drug effect analysis and by calculating the combination indexes (CIs) [16], after the cells were treated with equipotent doses of the two agents (50:50 cytotoxic ratio) for 96 hours. As shown in Table 2, we obtained consistent synergistic effects with low CIs (<0.9), which were calculated at 50% (CI_50_) or 75% (CI_75_) of cell lethality, in three out of four breast cancer cell lines (MCF-7, SKBR3, MDA-MB468); all HDACi were tested, and the best results were observed with VPA. The synergistic interaction was confirmed by an evaluation of the dose reduction indexes (DRIs), which represent the order of magnitude (i.e., fold change) of the dose reduction that was obtained for the IC_50_ (DRI_50_), in combination versus single-agent treatments (Table 2). Indeed, in all three cell lines, the combination resulted in the reduction of IC_50_ values (DRI_50_) from a mean of 1.46- to 4.58-fold for the HDACi, and from 2.44- to 14.5-fold for 5′-DFUR. Notably, in the breast non-tumorigenic cell line MCF-10A, in which VPA was unable to modulate the expression of TP, the combination of 5′-DFUR with either entinostat or VPA resulted in only additive (CIs = 0.9-1.1) if not antagonistic effect (CIs > 1.1). Moreover, the best interaction between 5′-DFUR and HDACi (CIs always < 0.7) was observed in MDA-MB468, which exhibits the lowest expression of TP out of any of the cell lines (Supplementary Figure S1). This again suggests the critical role of TP modulation in the observed synergism.

**Table 2:** Combination index (CI) and dose reduction index (DRI) values for HDACi (vorinostat, panobinostat, entinostat or VPA) and 5′-DFUR combination treatment

cell lines	^a^CI_50_ ± SD	^a^CI_75_ ± SD	^b^DRI at IC_50_ ± SD	
	**vorinostat + 5′DFUR**	**vorinostat + 5′DFUR**	**vorinostat**	**5′-DFUR**
**SKBR3**	0.63 ± 0.23	0.70 ± 0.21	3.42 ± 1.70	3.8 ± 1.82
**MDA-MB231**	0.98± 0.27	1.02± 0.1	3.46 ± 1.7	1.34 ± 0.62
**MDA-MB468**	0.49 ± 0.13	0.50 ± 0.14	2.64 ± 0.78	10.62 ± 4.1
**MCF-7**	0.71 ± 0.10	0.71 ± 0.068	2.67 ± 0.65	3 ± 1.10
	**panobinostat + 5′DFUR**	**panobinostat + 5′DFUR**	**panobinostat**	**5′-DFUR**
**SKBR3**	0.73 ± 0.15	0.71 ± 0.2	3.43 ± 1.76	3.44 ± 1.73
**MDA-MB231**	0.99 ± 0.4	0.93 ± 0.4	2.56 ± 1.07	2.17 ± 1.67
**MDA-MB468**	0.53 ± 0.2	0.58 ± 0.15	4.58 ± 2.7	5.45 ± 1.25
**MCF-7**	0.58 ± 0.16	0.61 ± 0.094	3.08 ± 1.72	4.5 ± 1.99
	**entinostat + 5′DFUR**	**entinostat + 5′DFUR**	**entinostat**	**5′-DFUR**
**SKBR3**	0.76 ± 0.01	0.74 ± 0.10	4.38 ± 2.28	2.44 ± 1.29
**MDA-MB231**	1.12 ± 0.04	1.26 ± 0.20	1.35 ± 0.24	2.92 ± 1.15
**MDA-MB468**	0.55 ± 0.045	0.54 ± 0.022	3.32 ± 1.56	3.0 ± 0.84
**MCF-7**	0.75 ± 0.16	0.76 ± 0.11	1.46 ± 0.36	14.5 ± 3.59
**MCF-10A**	1.22 ± 0.14	1.57 ± 0.036	1.53 ± 0.32	1.81 ± 0.10
	**VPA + 5′DFUR**	**VPA + 5′DFUR**	**VPA**	**5′-DFUR**
**SKBR3**	0.63 ± 0.14	0.59 ± 0.2	2.89 ± 1.5	4.45 ± 2.12
**MDA-MB231**	1.04 ± 0.037	1.06 ± 0.066	2.06 ± 0.36	1.71 ± 0.42
**MDA-MB468**	0.61 ± 0.17	0.65 ± 0.083	2.81 ± 1.27	5.81 ± 2.9
**MCF-7**	0.59 ± 0.24	0.56 ± 0.2	2.39 ± 0.76	6.45 ± 4.62
**MCF-10A**	1.11 ± 0.19	1.11 ± 0.18	0.99 ± 0.17	8.39 ± 7.13

a CI values (mean ± SD from at least three separate experiments performed in quadruplicates) computed at 50 and 75% of cell kill (CI_50_ and CI_75_, respectively) according by CalcuSyn software after 96 hours of treatment. Combinations were considered strongly synergistic when CIs were below 0.9.

b DRI values (mean ± SD from at least three separate experiments performed in quadruplicates) represents the order of magnitude (fold) of dose reduction obtained for IC_50_ (DRI_50_) in combination setting compared with each drug alone.

*Abbreviations:* VPA (valproic acid); 5′-DFUR (5′-deoxy-5-fluorouridine)

We found only additive antiproliferative effects in MDA-MB231 cells, even if we were able to modulate TP protein and mRNA levels by HDACi (Figure 1C, Supplementary Figure S2A, Supplementary Figure S4A and S4B). On this regard, we also investigate, by chromatin immunoprecipitation (ChIP) assay, the levels of two active histone marks at TP promoter: the acetylation of histone 3 lysine 9 (H3K9Ac) and the trimethylation of H3 lysine 4, (H3-K4me3). However, we observed a clear increase of both H3K9Ac and H3-K4me3 in cells treated with either VPA or VPA/5′-DFUR combination, in line with the TP protein and mRNA induction observed (Supplementary Figure S4C). Interestingly, we observed a slightly better effect (lower CIs) when we treated MDA-MB231 with higher relative doses of VPA (75:25 cytotoxic ratio, data not shown).

Finally, we confirm that TP modulation by low doses of VPA and by isoform-selective HDACi also translate in increase sensitivity to 5′-DFUR. In details, we demonstrated that combination treatment with IC_30_ of VPA resulted in up to 4.5-fold of 5′-DFUR IC_50_ reduction in MCF-7 cells (Supplementary Figure S5). Similarly, by combining 5′-DFUR with IC_30_ of RGFP-966 we obtained more than 2-fold of IC_50_ reduction. Conversely, a slight or no IC_50_ reduction was observed in combination with rocilinostat and MRLB-223, respectively.

### TP protein expression plays a critical role in synergistic antiproliferative and pro-apoptotic effect induced by the combination of VPA/5′-DFUR

**Figure 3:**
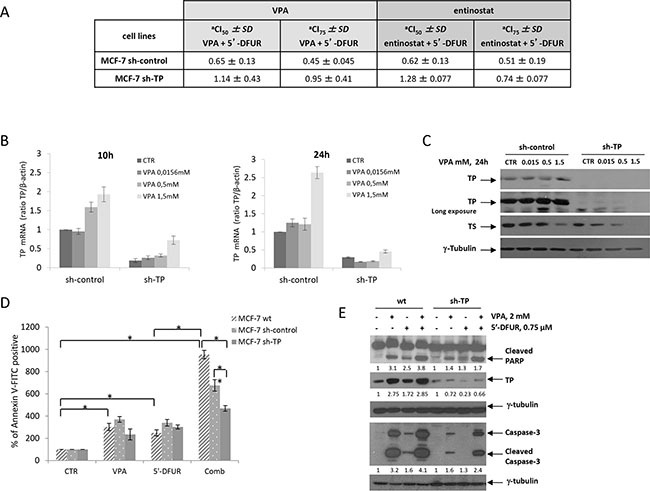
TP protein plays a critical role in the synergistic antiproliferative and pro-apoptotic effect induced by the combination of VPA and 5′-DFUR **A.** Synergistic interaction between VPA or entinostat and 5′-DFUR evaluated in MCF-7 sh-control and MCF-7 sh-TP cells by calculation of CI values when 50% and 75% of the cells had died (CI_50_ and CI_75_, respectively) according to CalcuSyn software after 96 hours of treatment (Mean ± SD from at least three separate experiments performed in quadruplicate). **B.** TP mRNA expression in MCF-7 sh-control and MCF-7 sh-TP cells that were either untreated or treated with increasing doses of VPA for 10 or 24 hours, as determined by qRealTime-PCR. **C.** TP and TS protein expression was determined by western blot in MCF-7 sh-control and MCF-7 sh-TP cells that were either untreated or treated with increasing doses of VPA for 24 hours. **D.** Apoptotic effect as assessed by flow cytometric analysis upon Annexin V-FITC staining of MCF-7 sh-control, MCF-7 wt, and MCF-7 sh-TP cells that were either untreated or treated for 72 hours with 2 mM VPA and/or 0.75 μM 5′-DFUR (Statistical analysis: One-way ANOVA, *p ≤ 0.001, **p = 0.003). **E.** The expression of cleaved-PARP, TP and cleaved-caspase 3 proteins as evaluated by western blot in MCF-7 wt and MCF-7 sh-TP cells that were untreated or treated for 48 hours with 2 mM VPA and/or 0.75 μM 5′-DFUR. γ-tubulin was used as the protein loading control.

To confirm that the HDACi-induced expression of TP is mechanistically correlated with the observed synergism, we next evaluated whether a stable TP-knockout by a specific shRNA would affect the antiproliferative synergistic effect of HDACi/5′-DFUR. MCF-7 cells were transfected with TP-specific sh-RNA or with control sh-RNA, and four pooled clones in which TP was stably silenced (MCF-7 sh-TP), clearly showed a significant reduction (5-fold) of TP transcript and protein, but similar TS expression compared with the empty vector-transfected clones (MCF-7 sh-control) or wild type (wt-) untrasfected MCF-7 cells (Supplementary Figure S6A). The loss of TP was able to reduce the antiproliferative effect observed after VPA/5′-DFUR combination (Supplementary Figure S6B). As shown in Figure 3B and 3C, increasing doses of VPA were unable to induce TP mRNA or protein expression in MCF-7 sh-TP cells compared with MCF-7 sh-control cells.

Combined treatment with either VPA or entinostat and 5′-DFUR for 96 hours at the same ratio as that evaluated in parental MCF-7 cells, resulted in synergistic antiproliferative effects in MCF-7 sh-control cells, but not in MCF-7 sh-TP cells as shown by the CI values (Figure 3A) and antiproliferative curves (Supplementary Figure S6B). Moreover, a statistically significant induction of apoptosis upon VPA/5′-DFUR combination treatment compared with single-agent treatments after 72 hours of exposure was observed in wt-MCF-7 cells but not in sh-TP cells, as demonstrated by Annexin V-FITC staining (Figure 3D). This effect correlates with a less pronounced amount of PARP (1.7-fold increase) and Caspase 3 (2.4-fold increase) cleavage, which was induced by combination treatment in sh-TP cells compared with wt-MCF7 (3.8-fold and 4.1-fold increase, respectively) (Figure 3E). Again, TP protein induction after VPA or VPA/5′-DFUR treatments was observed only in wt-MCF-7 cells but not in sh-TP cells (Figure 3E).

All together, these findings confirmed that TP up-regulation is critical for the synergistic antiproliferative effects and apoptotic cell death induced by the combination of VPA/5′-DFUR.

### *In vivo* synergistic antitumor effect of VPA in combination with capecitabine is limited to TP-expressing cells

**Figure 4:**
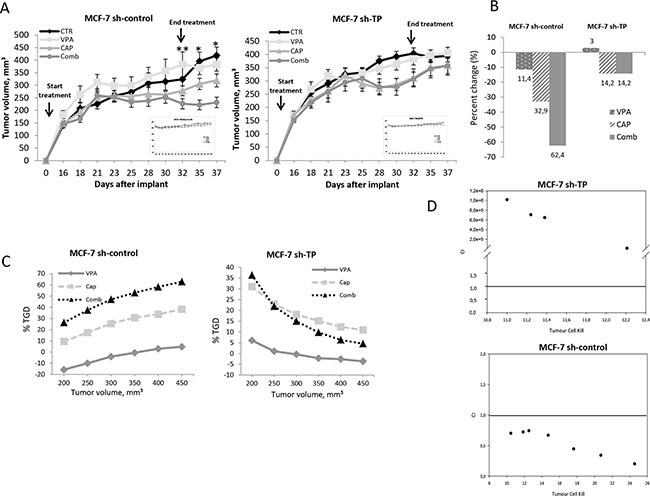
Synergistic antitumor effect induced by VPA/capecitabine combination in vivo strictly depends on TP modulation MCF-7 sh-control and MCF-7 sh-TP cells (8 × 10^6^) were s.c. injected into athymic mice as described in the Materials and Methods. When established tumors were palpable, mice were treated with VPA (200 mg/kg i.p.), capecitabine (359 mg/Kg p.o.), or both drugs 5 days/week for three weeks. **A.** Relative tumor volume curves for MCF-7 sh-control and MCF-7 sh-TP xenografts. Mean ± SD tumour volume measured at pre-specified time points (*n* = 7). *, *P* ≤ 0.001; and **, *P* ≤ 0.05. Inset, body weight measured three times/week. **B.** Tumour volume averages from each group at day 0 and day 37 were compared and presented as percentages of vehicle. **C.** Tumor growth delay (TGD), determined, in both MCF-7 sh-control and MCF-7 sh-TP xenografts, as %TGD = [(T − C) /C] × 100, where T and C are the mean times expressed in days for the treated or control groups, respectively, to reach a defined tumour volume (see Materials and Methods). **D.** *In vivo* VPA plus capecitabine combination studies evaluated by CalcuSyn in both MCF-7 sh-control and MCF-7 sh-TP xenografts. For the calculation of CI, the values of log cell kill (LCK) for a fixed tumour volume were considered (see Materials and Methods).

To confirm the synergistic interaction between HDACi and fluoropyrimidines *in vivo* and the critical role of HDACi-mediated TP up-regulation, we evaluated VPA in combination with capecitabine in both sh-control and sh-TP MCF-7 xenograft models in athymic mice. This was accomplished through the measurement of tumor volume (Figure 4A), the percent of tumor volume change (Figure 4B), the tumor growth delay (TGD) (Figure 4C) and the CI value (Figure 4D). Specifically, twenty-eight xenografted mice for each cell line were randomly assigned to receive sub-therapeutic doses of VPA (200 mg/kg i.p.), capecitabine (359 mg/Kg p.o.), both drugs in combination, or their respective vehicles as a control. Treatments were administered 5 days/week for three weeks.

As shown in Figure 4A, the combination treatment induced a significant inhibition of tumor growth compared with single-agent treatments only in the mice that were injected with control cells (sh-control MCF-7). In mice that were injected with sh-TP MCF-7 cells, no significant difference was observed compared with capecitabine treatment alone.

Moreover, by calculating the percent change in tumor volume from the time of initial treatment (day 0) to the end of the study (day 37), VPA, capecitabine and combination treatment reduced the tumor burden in sh-control mice by 11.4%, 32.9% and 62.4%, respectively. However, in the TP-knockout xenografts, VPA did not reduce the tumor burden, while capecitabine as both a single agent and in combination treatment, reduced the tumor burden by 14.2% (Figure 4B). The resultant TGD in the sh-control mice that were treated with a combination reached a peak of more than 60%, and the rate of tumor growth in the control, at that point, was 2-fold higher (Figure 4C). Either a reduced TGD or no effect was observed with single-agent capecitabine or VPA treatments, respectively. Furthermore, the synergistic effect of VPA/capecitabine was also confirmed by the reported CIs versus the LCK (Figure 4D). Notably, the evaluation of the TGD and CIs did not show any synergistic effect in TP-knockout xenografts (Figure 4C and 4D).

The combined treatment of VPA plus capecitabine was well tolerated, as shown by the maintenance of body weight (inset in Figure 4A) and the absence of other signs of acute or delayed toxicity in both xenograft models.

### Pharmacodynamic effects in xenograft tumors treated with a combination of VPA/capecitabine

At day 32, which represents the end of the treatment, we sacrificed one mouse per group (each with two tumors, one on each flank) to perform a pharmacodynamic analysis.

To confirm the synergistic effect and the modulation of both TS and TP in tumor cells *in vivo,* we isolated mRNA and proteins from tumor tissues and also performed IHC analysis on formalin fixed paraffin embedded (FFPE) tumor sections.

**Figure 5:**
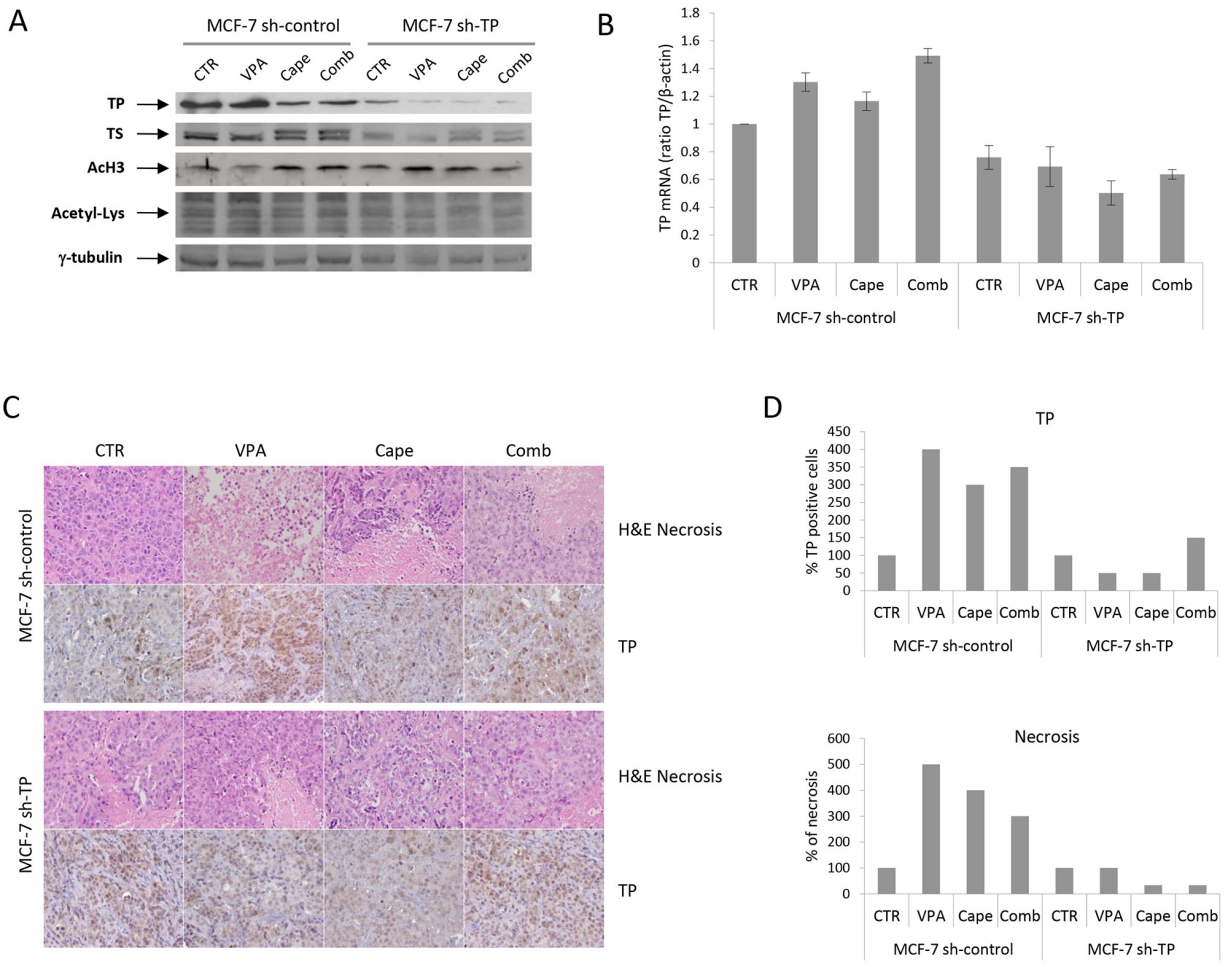
Role of TP expression on pharmacodynamic effects in xenograft tumors treated with VPA/capecitabine combination Tumors from one mouse (carrying two tumors, one on each flank) per group were collected at the end of the treatment in both MCF-7 sh-control and MCF-7 sh-TP xenografts. **A.** TP, TS, acetyl-H3 and acetyl-Lysine protein expression determined by western blot. γ-tubulin was used as protein loading control. **B.** TP mRNA expression determined by qRealTime-PCR. β-actin was used as housekeeping control gene to normalize qRealTime-PCR reactions. **C.** Paraffin-embedded tissues were generated for each group for hematoxylin and eosin stain (H&E) and immunohistochemistry analysis for TP as described in the Materials and Methods. Images were captured with a 20x or 40x objective on a light microscope. **D.** Tumour sections stained for TP were scored semiquantitatively for the percentage of positive cells. Necrotic area was evaluated as the percentage of necrosis inner to tumoral nodule.

As shown in Figure 5, the down-regulation of TS protein (Figure 5A) and the up-regulation of TP mRNA and protein levels (Figure 5B) after VPA and or combination treatment, were also observed in MCF-7 sh-control xenograft tumor tissues. MCF-7 sh-TP xenografts were confirmed to express very low levels of TP-protein or mRNA, which were not modulated by VPA. On the other hand, TS protein was down-regulated by VPA and by combination treatment in these tumors (Figure 5A). Similar data were obtained by an IHC analysis of xenograft tumor sections (Figure 5C and 5D). An analysis of mitotic and necrotic cells on H&E-stained slides of tumor sections demonstrated an increase in the percentage of necrotic cells after single-agent or combination treatment compared with vehicle treatment in MCF-7 sh-control xenografts but not in TP-knockout tumors (Figure 5C and 5D).

As expected, a western blot analysis of total lysine acetylation demonstrated a significant increase in both the VPA and VPA/capecitabine groups compared with controls, but we also observed an evident increase in histone-H3 acetylation in the capecitabine group (Figure 5A) as we have previously reported [18].

Finally, it was shown that TP, which is also known as pro-angiogenic platelet-derived endothelial cell growth factor (PD-ECGF), may contribute to angiogenesis through VEGF secretion and through other mechanisms [34]. However, we have not observed any modulation of VEGF mRNA expression (supplementary Figure S7A) or VEGF secretion (Supplementary Figure S7B) in VPA-treated cells. Similarly, no effect was observed in other angiogenesis-related factors such as PDGF, FGF, G-CSF and GM-CSF (Supplementary Figure S7C and S7D) or in any of the 27 cytokines that were analyzed (data not shown). Moreover, no significant induction of endothelial cells in histological tissue sections as evaluated by IHC analysis of CD31 expression was observed in VPA-treated tumors (Supplementary Figure S8).

Taken together, these data confirmed our *in vitro* findings, which demonstrate a synergistic antitumor interaction between VPA and capecitabine and the crucial role of TP up-regulation that occurs as a result of this effect.

## DISCUSSION

Although several improvements in terms of therapy have been made in the last years, the prognosis of patients with recurrent/metastatic breast cancer remains poor.

**Figure 6:**
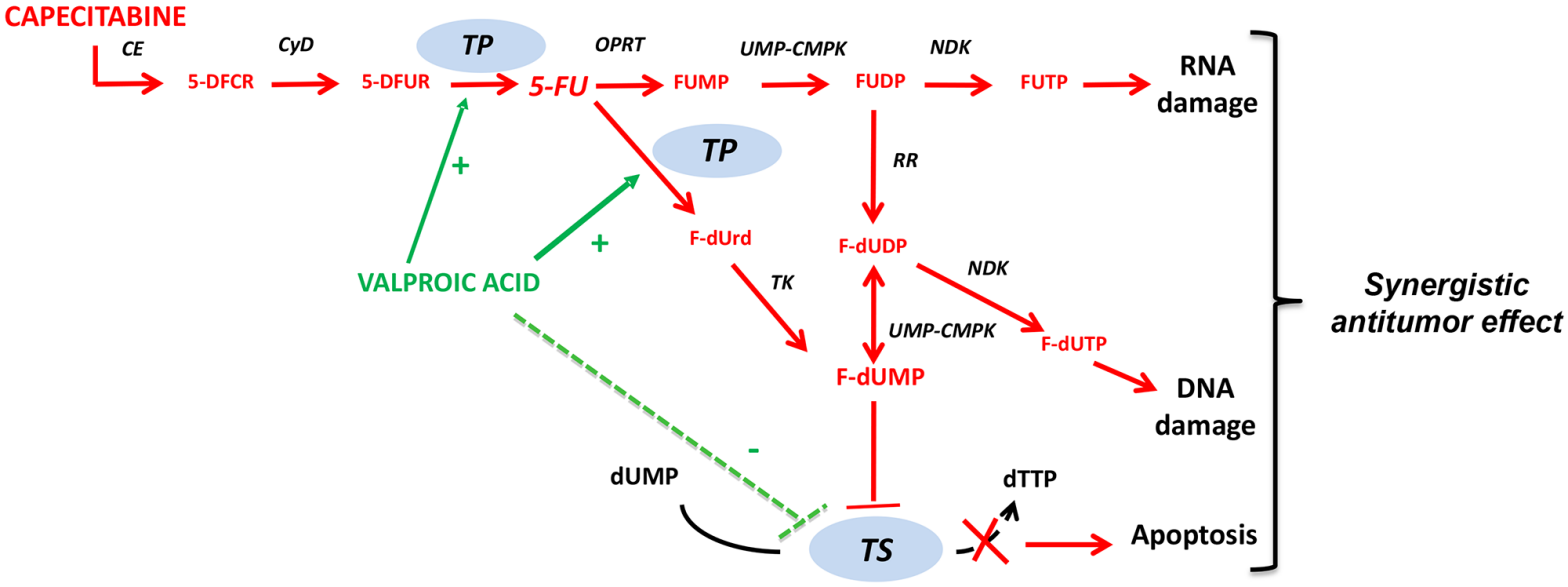
Mechanism of action of capecitabine and proposed synergistic interaction with valproic acid Capecitabine is an oral fluoropyrimidine absorbed unchanged through the gastrointestinal wall and converted to 5′-DFUR in the liver, by the sequential action of CE and CyD, and to 5-FU in tumor cells by TP. The active metabolite FdUMP by binding and inhibiting TS, causes rapid depletion of dTTP and thus thymine-less apoptosis. FdUTP and FUTP can be incorporated into DNA and RNA, respectively, contributing to 5-FU cytotoxicity. VPA synergizes with capecitabine preferentially by up-regulating TP, while down-regulation of TS expression is only partially involved. Abbreviation: CE, carboxylesterase; 5-DFCR, 5′-deoxy-5-fluorocytidine; CyD, cytidine deaminase; 5-DFUR, 5′-deoxy-5-fluorouridine; TP, thymidine phosphorylase; 5-FU, 5-fluorouracil; OPRT, orotate phosphoribosyl transferase; FUMP, 5-fluorouridine monophosphate; FUDP, 5-fluorouridinediphosphate; RR, ribonucleotide reductase; FUTP, 5-fluorouridine triphosphate; F-dUrd, 5-fluoro-2′-deoxyuridine; TK, thymidine kinase; F-dUDP, 5-Fluoro-2′-deoxyuridine diphosphate; F-dUMP, 5-fluoro-2′-deoxyuridine monophosphate; dUMP, 2′-deoxyuridine monophosphate; TS, thymidylate synthase; F-dUTP, 5-Fluoro-2′-deoxyuridine triphosphate; NDK, nucleoside diphosphate kinase; UMP-CMPK, urdine monophosphate-cytidine monophosphate kinase; dTTP, Deoxythymidine triphosphate.

In this study, we reported an innovative and rational therapeutic approach in breast cancer models based upon the combination of the HDACi VPA with capecitabine, a drug largely used to treat this disease. The results we obtained suggest that the up-regulation of TP, a critical enzyme in the final step of the metabolic transformation of the capecitabine metabolite 5′-DFUR to 5-FU, is induced by VPA and other HDACi. This up-regulation is crucial for the synergistic effect and may explain the mechanism behind the synergistic interaction between these two agents, which is observed both *in vitro* and *in vivo* (Figure 6).

Our results demonstrated *in vitro* that concurrent exposure of equipotent doses of an HDACi (e.g., VPA, vorinostat, panobinostat and entinostat) and 5′-DFUR, resulted in synergistic/additive antiproliferative and pro-apoptotic effects in all breast cancer cell lines examined and that these effects were independent of their characteristics and genetic backgrounds, being observed in ER+ MCF-7, HER-2 overexpressing SKBR3 or triple-negative MDA-468 cells. In MDA-MB231 cells, which have a hypermethylated TP promoter and express a low level of TP protein, we observed only an additive effect. However, in this cell line, both TP transcript and protein were up-regulated by all HDACi tested. We also performed a ChiP assay to measure the levels of two active histone marks at TP promoter and confirmed that they were both increase by VPA or VPA/5′-DFUR combination, in line with TP transcript and protein induction. Altogether, these results suggested that the absence of synergism in MDA-MB231 cells is not dependent on the lack of TP up-regulation. We can speculate that their different genetic background, being these cells the only one carrying both p53 and KRAS mutations, or their mesenchymal phenotype, could explain their intrinsic resistance to fluoropyrimidine, as suggested before [36, 37]. Alternatively, the presence of a mutated/inactive TP enzyme, described in disease other than cancer [38] could not be excluded and further studies are needed to investigate this hypothesis.

Although the synergistic intereraction with 5′-DFUR was observed with pan-HDACi such as vorinostat and panobinostat as well as with the class I selective HDACi such as entinostat and VPA, we focused on VPA for all subsequent experiments and for our *in vivo* study because, due to its safe use as a chronic therapy for epileptic disorders, it is a very good candidate for the translation of our findings in a proof-of-concept clinical study. Significantly, the synergistic effect demonstrated *in vitro* was confirmed *in vivo* in an MCF-7 breast cancer cell xenograft model where VPA plus capecitabine compared with single-agent treatments demonstrated a clear inhibition of tumor growth. This was confirmed by the assessment of the percentage of the reduction in the tumor volume, the TGD and the evaluation of the CI.

VPA up-regulates TP, both *in vitro* and *in vivo*, through a transcriptional mechanism, which confirmed the results obtained by our group in colorectal cancer models and those of Puppin *et al.* in breast cancer cells [18, 26].

Through the use of siRNA or isoform-specific HDACi, we provided several pieces of evidence that are consistent with the hypothesis that HDAC3, one of the direct targets of VPA, is the main isoform involved in the regulation of TP expression. Indeed, both HDAC3-specific siRNA and the isoform-specific inhibitor RGFP-966 are the most effective with respect to the induction of TP expression. This observation is clinically relevant because HDAC3 is emerging as a critical anticancer target [39–41] and more selective HDAC3 inhibitors may have a more favorable side-effect profile than class-I or non-selective HDACi. However, we cannot completely exclude the possibility that other class-I HDACs are involved. We observed that the HDAC6-specific siRNA and the HDAC6-inhibitor rocilinostat also up-regulated TP expression. However the observation that rocilinostat could target also class-I HDACs [31] and that tubacin, another HDAC6-inhibitor, only slightly affect TP expression, suggest that TP regulation could be confined to class-I HDACs. Significantly, HDAC3 inhibitor RGFP-966 in combination treatment induced a 2-fold reduction of 5′-DFUR IC_50_ compared to modest or no effect obtained with HDAC6 inhibitor rocilinostat or HDAC1-2 inhibitor MRLB-223. Moreover, the compensatory modulation of other HDAC isoforms upon siRNA-mediated HDAC6 down-modulation, could, at least in part, explain some of our contradictory findings. Furthermore, the evident up-regulation of TP by HDACi (i.e., such as by VPA, depsipeptide or entinostat) not supposed to affect HDAC6, reinforces our hypothesis. Nevertheless, further studies are necessary to confirm the specific involvement of HDAC3 in the regulation of TP expression in cancer cells.

Low doses of VPA that are typically given as anticonvulsant therapy in the treatment of epileptic patients and that correspond to the plasma level range of 50-100 mg/ml, are included within the clinical limits of tolerance. Remarkably, these low doses were able to significantly modulate TP expression within a few hours. This suggests that the enhanced lethality of combination treatment could primarily be attributed to this mechanism. Indeed low doses of VPA were able to reduce 5′-DFUR IC_50_ up to 4.5-fold in combination treatment. Moreover, we showed that TP-knockout in MCF-7 cells significantly suppresses both *in vitro* and *in vivo*, the synergistic antitumor effect induced by the VPA/capecitabine combination, which confirms the critical role of TP in the observed synergism.

We also showed that VPA in breast cancer cells and in xenograft tumors induced the down-regulation of TS, the target of 5-FU. However, although this effect may contribute to the synergistic antitumor effect of fluoropyrimidine, it is not essential (Figure 6). Indeed, in TP-knockout cells, even though we observed TS down-regulation upon VPA treatment, both *in vitro* and *in vivo*, the synergistic antitumor effect with capecitabine was abrogated.

Interestingly, in the non-tumorigenic mammary cell line MCF-10A, we did not observe TP and TS modulation after VPA treatment or the synergistic/additive effect between VPA or entinostat and 5′-DFUR. Consequently, we can assume that, as demonstrated for other genes, TP can be specifically modulated by HDACi in tumor cells in which TP is deregulated either by promoter methylation or other mechanisms, while normal cells are not affected. This observation suggests that the increased antitumor effect observed in tumor cells both *in vitro* and *in vivo* by a combination of HDACi and fluoropyrimidines should not translate into an increased toxicity in normal cells. Indeed, no additional toxic effect was observed *in vivo* in the setting of combination therapy compared with single-agent treatments.

PD-ECGF/TP expression may contribute to angiogenesis, tumor progression and metastasis via mechanisms that remain to be defined [34]. Interestingly, we did not detect any modulation of angiogenesis either *in vitro* or *in vivo* upon VPA treatment. This is in agreement with several reports that have demonstrated that HDACi including VPA prevent tumor invasion and metastasis as well as tumor-induced angiogenesis [42].

Several preclinical and clinical indications have demonstrated that intrinsic or acquired resistance to fluoropyrimidines correlates with low or deficient intratumoral TP expression [6, 7, 43, 44], which suggests that an increase in TP expression might enhance the sensitivity of tumor cells to these drugs. In this regard, when combined with capecitabine, several chemotherapeutics such as taxanes and cyclophosphamide, X-ray irradiation or targeted therapy such as the EGFR tyrosine kinase inhibitor gefitinib, have shown synergistic antitumor effects via the induction of TP up-regulation [34, 45–47]. Indeed, TP-inducible chemotherapeutics such as taxanes in combination with or followed by capecitabine, showed an increased response rate, time to progression and survival in patients with breast cancer and TP expression has been suggested to be a predictive marker of therapeutic benefit [4, 7, 48, 49]. Anthracycline- or taxane-based regimens are commonly used in the treatment of breast cancer as first-line in the metastatic settings. However, there is no single accepted standard of care after failure of anthracycline and taxane therapy. Capecitabine is widely considered a drug with a favourable risk:benefit profile in patients with metastatic breast cancer, being used in the first-, second-, and third-line settings as single agent or in combination with several other drugs. Capecitabine has also been the control arm in several phase III trials in metastatic setting. Recently the phase III study leading to the approval of Eribulin mesylate (eribulin), a novel microtubule dynamics inhibitor, as single agent in metastatic breast cancer, demonstrated that eribulin was not shown to be superior to capecitabine with regard to overall survival, progression-free survival or health-related quality of life [50, 51]. Overall, although several novel drugs are currently available, capecitabine remains one of the most effective and used drugs in the treatment of metastatic breast cancer [52].

In the present study, we showed for the first time the synergistic interaction between capecitabine and VPA, which are two generic drugs that can be administered orally with consequent increased compliance for the patients, providing a rationale for the evaluation of the clinical efficacy of VPA/capecitabine combination in breast cancer patients.

Based on the current study and on the results that were obtained in colorectal cancer models, in which we showed a synergistic antitumor effect of the HDACi vorinostat and capecitabine, we recently launched a phase-I/II clinical study (V-ShoRT-R3 trial) to explore whether the addition of both VPA and capecitabine to short-course radiotherapy, before optimal radical surgery, might increase the pathologic complete tumor regression rate in low-moderate risk rectal cancer patients [53].

## MATERIALS AND METHODS

### Reagents

Valproic acid (VPA) was purchased from Enzo Life Sciences and 5′-deoxy-5-fluorouridine (5′-DFUR) was purchased from Sigma-Aldrich. Stock solutions were prepared in sterile water and diluted to appropriate concentrations in culture medium before addition to the cells. Entinostat (MS-275) was from Adipogen, panobinostat (LBH589) from Biovision Incorporated, trichostatin A (TSA) from Alexis Biochemicals, tubacin and niltubacin were kindly provided by Dr. S.L. Schreiber and Dr. Ralph Mazitschek. Vorinostat was from Calbiochem, RGFP966, romidepsin and rocilinostat were from Selleck Chemicals and MRLB223 was kindly provided by Dr S. Minucci. Stock solutions were prepared in DMSO. Capecitabine (Xeloda) was from Roche. Capecitabine is a prodrug that needs the Carboxyl esterase activity to be converted in the first metabolic step, but, due to his low level expression in most cancer cell lines, all *in vitro* studies in cancer cells were performed with capecitabine-metabolite 5′-DFUR, which requires the presence of TP to be converted into the active 5-FU drug, as previously described [18].

All media, serum, antibiotics, and glutamine were from Lonza.

Primary antibodies (Abs) for western blotting: thymidylate synthase (TS)-Ab (Rockand Immunochemicals Inc.; cod. 100-601-199), E-cadherin-Ab (Abcam, cod. ab40772), vimentin-Ab (Dako; cod. M0725), poly-(ADP-ribose)-polymerase (PARP)-Ab (BD Biosciences, cod. 556494), acetyl-α-tubulin-Ab (Sigma-Aldrich, cod. T7451), γ-Tubulin-Ab (cod. sc-7396) and thymidine phosphorylase (TP)/platelet-derived endothelial growth factor (PD-ECGF)-Ab (cod. sc-71867) (Santa Cruz Biotechnology), GAPDH-Ab (cod. #2118), α-tubulin-Ab (cod. #2144), acetylated-lysine monoclonal-Ab (cod. #9681), acetyl-H3-Ab (cod. #9649) and caspase-3-Ab (cod. #9661) (Cell Signalling Technology). For IHC mouse monoclonal TP/PD-ECGF-Ab (Thermo Scientific; cod. MS-499) or goat polyclonal CD31/Pecam-1-Ab (Santa Cruz; cod. sc-1506) were used. Actinomycin D is from Sigma Aldrich.

### Cell culture conditions

The MCF-7 and SKBR3 cells were maintained in Dulbecco's modified Eagle's medium (DMEM), whereas MDA-MB468 were maintained in DMEM/F12 medium and MDA-MB231 in RPMI-1640 medium. All media were supplemented with 10% heat-inactivated fetal bovine serum, 50 units/mL penicillin, 500 μg/mL streptomycin, and 4 mmol/L glutamine. MCF-10A cells were maintained in Mammary Epithelial Cell Growth Medium (MEGM™). All cell lines were cultivated at 37°C in a humidified 5% CO_2_ atmosphere. All cell lines were from American Type Culture Collection, were regularly inspected for mycoplasma and have been authenticated with a short-tandem repeat profile according to LGC Standards.

### Cell viability assay and *in vitro* drug combination studies

Cell viability/proliferation was evaluated by a spectrophotometric dye incorporation assay using sulforhodamine B (SRB, ICN Biomedicals) in quadruplicate in 96-well plates, 96 hours after treatment, as described previously [16].

Drug combination studies were based on concentration-effect curves generated as a plot of the fraction of unaffected (i.e., surviving) cells versus the drug concentration [18]. Serial dilutions of equipotent doses of the two agents in combination were tested. Synergism, additivity, and antagonism were quantified after an evaluation of the combination index (CI), which was calculated by the Chou-Talalay equation with CalcuSyn software (Biosoft), as described elsewhere [54]. A CI < 0.9, CI = 0.9–1.2, and CI > 1.2 indicated a synergistic, an additive or an antagonistic effect, respectively [16]. The DRI (dose reduction index) determines the magnitude of dose reduction allowed for each drug when given in combination, compared with the concentration of a single agent that is needed to achieve the same effect [18].

### Western blot analysis

Immunoblotting was performed as described elsewhere [54]. Densitometric analysis was performed by NIH ImageJ software.

### Gene-knockout

RNA interference (siRNA) against human HDAC1, HDAC3, HDAC6, scrambled RNAi used as the negative control (Integrated DNA Technologies), or HDAC2 (Ambion Life Technologies) were transiently transfected into MCF-7 cells using Lipofectamine 2000 (Invitrogen) according to the manufacturer's instructions.

TP-knockout was accomplished by the transfection of MCF-7 cells via Lipofectamine 2000 with a vector in a pGFP-V-RS plasmid that carries a short hairpin RNA (shRNA) that targets human TP mRNA. Four stable clones selected by puromycin resistance were pooled together (MCF-7 sh-TP). Scrambled transfected cells were also selected as a control (MCF-7 sh-control).

### Real-time quantitative PCR and mRNA stability assay

Total RNA was extracted from the cells by Trizol reagent according to the manufacturer's instructions (Invitrogen). The reverse transcription-PCR (RT-PCR) assay was performed with a High-Capacity cDNA Reverse-Transcription Kit (Applied Biosystems). TS (Hs00426586_m1), TP (Hs00157317_m1), VEGFa (Hs00900055_m1), HDAC2 (Hs00231032_m1) and GADD45 (Hs99999173_m1) mRNA expression was quantified as previously described [18]. Each gene was tested in each cell line in triplicates in three independent experiments. The relative changes in gene expression were normalized to endogenous β-actin levels by the −2^ΔΔCT^ method [18]. HDAC1 (QT00015239, Qiagen), HDAC3 (QT00093730) and HDAC6 (QT 00002709) mRNA expression levels were quantified by the fluorescent dye SYBR-Green-I method (Qiagen) and were normalized to endogenous β-actin (QT00095431) levels.

To study the effect of VPA on TP mRNA stability, MCF-7 cells were untreated or treated with 1 μg/ml actinomycin D and/or VPA (2.5mM), for 10 hours. TP and GADD45a mRNA expression was determined as described above.

### Chromatin immunoprecipitation assay

MDA-MB231 cells (5 × 10^6^) treated for 24 hours with the indicated drugs were fixed with 1% formaldehyde. After cross-linking, crude nuclear extracts were isolated and subjected to sonication, which resulted in 200 bp to 500 bp DNA fragments. After immunoprecipitation with the anti-H3K4me3 (Abcam cod. ab8580) or anti-H3K9/14Ac (Millipore cod. 06-599) antibodies (2μg/100μg of nuclear lysate), the immunocomplexes were purified by co-precipitation with protein A-Sepharose (GE Healthcare). Species matched IgG were used as the negative control. The amount of recovered DNA was determined, and the quantification of chromatin-immunoprecipitated DNA fragments was performed using qRT-PCR with the following primers: TYMP forward (GAGGCAGACACGGACGAG) and TYMP reverse (GGTCATCAAGGCTGCCATC) specific for the TYMP promoter region chr-22: 50, 529, 694-50, 529, 866. The enrichment of the DNA was calculated in terms of % input = 2^−ΔCt^ × 100, where ΔCt (threshold cycle) was determined by Ct_IP sample_ - Ct_Input_, and 100 refers to the input being 1% of the chromatin amount exposed to the immunoprecipitation.

### Detection of apoptosis

Apoptosis was measured by flow cytometry using the Annexin V-FITC, as described previously [55].

### Cytokine assay

Vascular endothelial growth factor (VEGF), Platelet-Derived Growth Factor (PDGF), Fibroblast growth factor (FGF), Granulocyte colony-stimulating factor (G-CSF) and Granulocyte-macrophage colony-stimulating factor (GM-CSF) and other cytokine levels in MCF-7 cell supernatants were assayed using Bio-Plex Pro Human Cytokine 27-plex Assay (Biorad) and acquired on a Bio-Plex array reader (Luminex) according to the manufacturer's instructions [35].

### *In vivo* xenograft studies

Female NOD/SCID athymic mice (Harlan) were acclimatized in the Animal Care Facility of Biogem S.C.A.R.L. in accordance with the institutional guidelines of the Italian Ministry of Health Animal Care and Use Committee, before they were injected subcutaneously (*s.c*.) with a 60-day release 17β-estradiol pellet (Innovative Research of America). After three days, MCF-7 sh-control or MCF-7 sh-TP cells (8 × 10^6^) diluted in 200 ml [M199 medium/Matrigel GF (Becton Dickinson) 1/1] were injected *s.c* in both flank regions of the mice. When the tumors became palpable, the mice were randomized into four experimental groups (*n* = 7) for each cell line. Based on pilot studies (data not shown) and previous reports [18], the mice were treated intraperitoneally (*i.p.)* with VPA (200 mg/Kg melted in water and diluted in a physiological solution) in the morning and/or capecitabine *per os* (359 mg/Kg in 40 mM citrate buffer-pH 6 containing 5% Arabic gum) in the afternoon 5 days/week for three weeks. Mice in the control group were treated with both physiological solution and citrate buffer/Arabic gum vehicles. Tumor volume (TV) (mm3) was calculated by the formula TV=[length (mm) × width (mm)^2^]/2, where the width and the length are the shortest and the longest diameters, respectively, as measured by a caliper three times/week. The mice were monitored daily for clinical signs and mortality; in addition, body weight recordings were performed triweekly. At the end of the study, at day 37 after the implantation and one week after the end of treatment, the mice were sacrificed by cervical dislocation. Tumor growth delay (TGD) was determined as %TGD = [(T − C)/C] × 100, where T and C are the mean times in days required to reach the same fixed tumor volume in the treated group and control group, respectively [54]. The percent change was calculated for all experimental groups using the formula: [(Day37 tumor volume – Day0 tumor volume)/Day0 tumor volume × 100]. The percent change in the experimental groups was compared with that of the vehicle control group using the equation: [(Overall percent change experimental – overall percent change vehicle)/overall percent change vehicle × 100] [56]. For the calculation of CI by CalcuSyn, the values of Cell Kill (CK) for a fixed tumor volume were considered [determined by the log CK (LCK)]. Log Cell Kill was determined by LCK = (T-C)/(3.3-T_d_), where T and C are the same values as described above; T_d_ represents the mean control group doubling time required to reach a fixed tumor volume and is expressed in days [54].

### Histology, immunohistochemistry, western blotting and Real-Time PCR analysis from tumor samples

At the end of treatment, one mouse per group was sacrificed for a pharmacodynamic study, and tumors (one on each flank) were collected; half of the tumors were stored at −80°C while the other half were fixed in 10% buffered formalin and embedded in paraffin for immunohistochemical analysis.

Protein and mRNA expression levels of the tumor samples were evaluated as previously described.

We evaluated necrosis by hematoxylin and eosin (H&E) stain, and the expression of TP and CD31/Pecam-1 by immunohistochemistry (IHC). Briefly, the sections were incubated with primary antibodies and then with biotin-conjugated secondary antibodies before incubation with specific Streptavidin HRP-conjugated tertiary antibody (Dako). Peroxidase reactivity was visualized using a 3,3′-diaminobenzidine (Abcam). A single pathologist (R.F.) performed a blinded analysis of the slides.

### Statistical analysis

The result of the *in vitro* cell viability assay is expressed as the mean for at least three independent experiments, which were conducted in quadruplicate (±SD). The results of the apoptotic analysis are expressed as the mean for at least three independent experiments (±SD), and the statistical significance was determined by one-way ANOVA and Holm-Sidak methods. A *p* < 0.05 was considered to be statistically significant. Representative results from a single experiment of qReal-Time PCR, western blot and immunohistochemistry are presented; additional experiments yielded similar results.

Statistical significance in the differences of tumor growth *in vivo* was determined by the one-way ANOVA and Tukey Test (*p* < 0.05). All statistical evaluations were performed with Sigma Stat software (Systat Software Inc).

## SUPPLEMENTARY FIGURES



## Footnotes

1 http://www.fda.gov/newsevents/newsroom/pressannouncements/ucm403929.htm

2 http://www.fda.gov/NewsEvents/Newsroom/PressAnnouncements/ucm435296.htm

## References

[1] Siegel RL, Miller KD, Jemal A (2015). Cancer statistics, 2015. CA.

[2] Daniele G, Gallo M, Piccirillo MC, Giordano P, D'Alessio A, Del Giudice A, La Porta ML, Perrone F, Normanno N, De Luca A (2013). Pharmacokinetic evaluation of capecitabine in breast cancer. Expert opinion on drug metabolism & toxicology.

[3] Beslija S, Bonneterre J, Burstein HJ, Cocquyt V, Gnant M, Heinemann V, Jassem J, Kostler WJ, Krainer M, Menard S, Petit T, Petruzelka L, Possinger K (2009). Third consensus on medical treatment of metastatic breast cancer. Annals of oncology.

[4] Gligorov J, Doval D, Bines J, Alba E, Cortes P, Pierga JY, Gupta V, Costa R, Srock S, de Ducla S, Freudensprung U, Mustacchi G (2014). Maintenance capecitabine and bevacizumab versus bevacizumab alone after initial first-line bevacizumab and docetaxel for patients with HER2-negative metastatic breast cancer (IMELDA): a randomised, open-label, phase 3 trial. Lancet Oncol.

[5] Longley DB, Harkin DP, Johnston PG (2003). 5-fluorouracil: mechanisms of action and clinical strategies. Nat Rev Cancer.

[6] Patterson AV, Zhang H, Moghaddam A, Bicknell R, Talbot DC, Stratford IJ, Harris AL (1995). Increased sensitivity to the prodrug 5′-deoxy-5-fluorouridine and modulation of 5-fluoro-2′-deoxyuridine sensitivity in MCF-7 cells transfected with thymidine phosphorylase. Br J Cancer.

[7] Bonotto M, Bozza C, Di Loreto C, Osa EO, Poletto E, Puglisi F (2013). Making capecitabine targeted therapy for breast cancer: which is the role of thymidine phosphorylase?. Clinical breast cancer.

[8] Takagi K, Miki Y, Nakamura Y, Hirakawa H, Kakugawa Y, Amano G, Watanabe M, Ishida T, Sasano H, Suzuki T (2015). Immunolocalization of thymidylate synthase as a favorable prognostic marker in estrogen receptor-positive breast carcinoma. Histology and histopathology.

[9] Budillon A, Di Gennaro E, Bruzzese F, Rocco M, Manzo G, Caraglia M (2007). Histone deacetylase inhibitors: a new wave of molecular targeted anticancer agents. Recent Pat Anticancer Drug Discov.

[10] Mann BS, Johnson JR, Cohen MH, Justice R, Pazdur R (2007). FDA approval summary: vorinostat for treatment of advanced primary cutaneous T-cell lymphoma. The oncologist.

[11] Khot A, Dickinson M, Prince HM (2013). Romidepsin for peripheral T-cell lymphoma. Expert review of hematology.

[12] Lee HZ, Kwitkowski VE, Del Valle PL, Ricci MS, Saber H, Habtemariam BA, Bullock J, Bloomquist E, Li Shen Y, Chen XH, Brown J, Mehrotra N, Dorff S (2015). FDA Approval: Belinostat for the Treatment of Patients with Relapsed or Refractory Peripheral T-cell Lymphoma. Clin Cancer Res.

[13] (2015). Panobinostat approved for multiple myeloma. Cancer discovery.

[14] Raha P, Thomas S, Munster PN (2011). Epigenetic modulation: a novel therapeutic target for overcoming hormonal therapy resistance. Epigenomics.

[15] Yardley DA, Ismail-Khan RR, Melichar B, Lichinitser M, Munster PN, Klein PM, Cruickshank S, Miller KD, Lee MJ, Trepel JB (2013). Randomized phase II, double-blind, placebo-controlled study of exemestane with or without entinostat in postmenopausal women with locally recurrent or metastatic estrogen receptor-positive breast cancer progressing on treatment with a nonsteroidal aromatase inhibitor. J Clin Oncol.

[16] Di Gennaro E, Bruzzese F, Pepe S, Leone A, Delrio P, Subbarayan PR, Avallone A, Budillon A (2009). Modulation of thymidilate synthase and p53 expression by HDAC inhibitor vorinostat resulted in synergistic antitumor effect in combination with 5FU or raltitrexed. Cancer Biol Ther.

[17] Bruzzese F, Rocco M, Castelli S, Di Gennaro E, Desideri A, Budillon A (2009). Synergistic antitumor effect between vorinostat and topotecan in small cell lung cancer cells is mediated by generation of reactive oxygen species and DNA damage-induced apoptosis. Molecular cancer therapeutics.

[18] Di Gennaro E, Piro G, Chianese MI, Franco R, Di Cintio A, Moccia T, Luciano A, de Ruggiero I, Bruzzese F, Avallone A, Arra C, Budillon A (2010). Vorinostat synergises with capecitabine through upregulation of thymidine phosphorylase. British journal of cancer.

[19] Bruzzese F, Leone A, Rocco M, Carbone C, Piro G, Caraglia M, Di Gennaro E, Budillon A (2011). HDAC inhibitor vorinostat enhances the antitumor effect of gefitinib in squamous cell carcinoma of head and neck by modulating ErbB receptor expression and reverting EMT. J Cell Physiol.

[20] Chateauvieux S, Morceau F, Dicato M, Diederich M (2010). Molecular and therapeutic potential and toxicity of valproic acid. J Biomed Biotechnol.

[21] Munster P, Marchion D, Bicaku E, Schmitt M, Lee JH, DeConti R, Simon G, Fishman M, Minton S, Garrett C, Chiappori A, Lush R, Sullivan D (2007). Phase I trial of histone deacetylase inhibition by valproic acid followed by the topoisomerase II inhibitor epirubicin in advanced solid tumors: a clinical and translational study. J Clin Oncol.

[22] Atmaca A, Al-Batran SE, Maurer A, Neumann A, Heinzel T, Hentsch B, Schwarz SE, Hovelmann S, Gottlicher M, Knuth A, Jager E (2007). Valproic acid (VPA) in patients with refractory advanced cancer: a dose escalating phase I clinical trial. Br J Cancer.

[23] Arce C, Perez-Plasencia C, Gonzalez-Fierro A, de la Cruz-Hernandez E, Revilla-Vazquez A, Chavez-Blanco A, Trejo-Becerril C, Perez-Cardenas E, Taja-Chayeb L, Bargallo E, Villarreal P, Ramirez T, Vela T (2006). A proof-of-principle study of epigenetic therapy added to neoadjuvant doxorubicin cyclophosphamide for locally advanced breast cancer. PloS one.

[24] Munster P, Marchion D, Bicaku E, Lacevic M, Kim J, Centeno B, Daud A, Neuger A, Minton S, Sullivan D (2009). Clinical and biological effects of valproic acid as a histone deacetylase inhibitor on tumor and surrogate tissues: phase I/II trial of valproic acid and epirubicin/FEC. Clin Cancer Res.

[25] Fazzone W, Wilson PM, Labonte MJ, Lenz HJ, Ladner RD (2009). Histone deacetylase inhibitors suppress thymidylate synthase gene expression and synergize with the fluoropyrimidines in colon cancer cells. Int J Cancer.

[26] Puppin C, Puglisi F, Pandolfi M, Di Loreto C, Damante G (2011). Histone deacetylase inhibitors induce thymidine phosphorylase expression in cultured breast cancer cell lines. Oncol Rep.

[27] Haggarty SJ, Koeller KM, Wong JC, Grozinger CM, Schreiber SL (2003). Domain-selective small-molecule inhibitor of histone deacetylase 6 (HDAC6)-mediated tubulin deacetylation. Proc Natl Acad Sci U S A.

[28] Thurn KT, Thomas S, Raha P, Qureshi I, Munster PN (2013). Histone deacetylase regulation of ATM-mediated DNA damage signaling. Mol Cancer Ther.

[29] Bradner JE, West N, Grachan ML, Greenberg EF, Haggarty SJ, Warnow T, Mazitschek R (2010). Chemical phylogenetics of histone deacetylases. Nature chemical biology.

[30] Newbold A, Matthews GM, Bots M, Cluse LA, Clarke CJ, Banks KM, Cullinane C, Bolden JE, Christiansen AJ, Dickins RA, Miccolo C, Chiocca S, Kral AM (2013). Molecular and biologic analysis of histone deacetylase inhibitors with diverse specificities. Molecular cancer therapeutics.

[31] Thaler F, Mercurio C (2014). Towards selective inhibition of histone deacetylase isoforms: what has been achieved, where we are and what will be next. ChemMedChem.

[32] Santo L, Hideshima T, Kung AL, Tseng JC, Tamang D, Yang M, Jarpe M, van Duzer JH, Mazitschek R, Ogier WC, Cirstea D, Rodig S, Eda H (2012). Preclinical activity, pharmacodynamic, and pharmacokinetic properties of a selective HDAC6 inhibitor, ACY-1215, in combination with bortezomib in multiple myeloma. Blood.

[33] Wells CE, Bhaskara S, Stengel KR, Zhao Y, Sirbu B, Chagot B, Cortez D, Khabele D, Chazin WJ, Cooper A, Jacques V, Rusche J, Eischen CM (2013). Inhibition of histone deacetylase 3 causes replication stress in cutaneous T cell lymphoma. PloS one.

[34] Liekens S, Bronckaers A, Perez-Perez MJ, Balzarini J (2007). Targeting platelet-derived endothelial cell growth factor/thymidine phosphorylase for cancer therapy. Biochem Pharmacol.

[35] Milone MR, Pucci B, Bruzzese F, Carbone C, Piro G, Costantini S, Capone F, Leone A, Di Gennaro E, Caraglia M, Budillon A (2013). Acquired resistance to zoledronic acid and the parallel acquisition of an aggressive phenotype are mediated by p38-MAP kinase activation in prostate cancer cells. Cell death & disease.

[36] Zhang W, Feng M, Zheng G, Chen Y, Wang X, Pen B, Yin J, Yu Y, He Z (2012). Chemoresistance to 5-fluorouracil induces epithelial-mesenchymal transition via up-regulation of Snail in MCF7 human breast cancer cells. Biochem Biophys Res Commun.

[37] Guarcello V, Blanquicett C, Naguib FN, El Kouni MH (2008). Suppression of thymidine phosphorylase expression by promoter methylation in human cancer cells lacking enzyme activity. Cancer Chemother Pharmacol.

[38] Slama A, Lacroix C, Plante-Bordeneuve V, Lombes A, Conti M, Reimund JM, Auxenfants E, Crenn P, Laforet P, Joannard A, Seguy D, Pillant H, Joly P (2005). Thymidine phosphorylase gene mutations in patients with mitochondrial neurogastrointestinal encephalomyopathy syndrome. Molecular genetics and metabolism.

[39] Muller BM, Jana L, Kasajima A, Lehmann A, Prinzler J, Budczies J, Winzer KJ, Dietel M, Weichert W, Denkert C (2013). Differential expression of histone deacetylases HDAC1, 2 and 3 in human breast cancer--overexpression of HDAC2 and HDAC3 is associated with clinicopathological indicators of disease progression. BMC cancer.

[40] Spurling CC, Godman CA, Noonan EJ, Rasmussen TP, Rosenberg DW, Giardina C (2008). HDAC3 overexpression and colon cancer cell proliferation and differentiation. Molecular carcinogenesis.

[41] Minami J, Suzuki R, Mazitschek R, Gorgun G, Ghosh B, Cirstea D, Hu Y, Mimura N, Ohguchi H, Cottini F, Jakubikova J, Munshi NC, Haggarty SJ (2014). Histone deacetylase 3 as a novel therapeutic target in multiple myeloma. Leukemia.

[42] Shiva Shankar TV, Willems L (2014). Epigenetic modulators mitigate angiogenesis through a complex transcriptomic network. Vascular pharmacology.

[43] de Bruin M, van Capel T, Van der Born K, Kruyt FA, Fukushima M, Hoekman K, Pinedo HM, Peters GJ (2003). Role of platelet-derived endothelial cell growth factor/thymidine phosphorylase in fluoropyrimidine sensitivity. Br J Cancer.

[44] Salonga D, Danenberg KD, Johnson M, Metzger R, Groshen S, Tsao-Wei DD, Lenz HJ, Leichman CG, Leichman L, Diasio RB, Danenberg PV (2000). Colorectal tumors responding to 5-fluorouracil have low gene expression levels of dihydropyrimidine dehydrogenase, thymidylate synthase, and thymidine phosphorylase. Clin Cancer Res.

[45] Sawada N, Ishikawa T, Sekiguchi F, Tanaka Y, Ishitsuka H (1999). X-ray irradiation induces thymidine phosphorylase and enhances the efficacy of capecitabine (Xeloda) in human cancer xenografts. Clin Cancer Res.

[46] Endo M, Shinbori N, Fukase Y, Sawada N, Ishikawa T, Ishitsuka H, Tanaka Y (1999). Induction of thymidine phosphorylase expression and enhancement of efficacy of capecitabine or 5′-deoxy-5-fluorouridine by cyclophosphamide in mammary tumor models. Int J Cancer.

[47] Ait-Tihyaty M, Rachid Z, Mihalcioiu C, Jean-Claude BJ (2012). Inhibition of EGFR phosphorylation in a panel of human breast cancer cells correlates with synergistic interactions between gefitinib and 5′-DFUR, the bioactive metabolite of Xeloda. Breast cancer research and treatment.

[48] Puglisi F, Cardellino GG, Crivellari D, Di Loreto C, Magri MD, Minisini AM, Mansutti M, Andreetta C, Russo S, Lombardi D, Perin T, Damante G, Veronesi A (2008). Thymidine phosphorylase expression is associated with time to progression in patients receiving low-dose, docetaxel-modulated capecitabine for metastatic breast cancer. Annals of oncology.

[49] Bonotto M, Fontanella C, Puglisi F (2015). Looking for predictive markers in breast cancer. Lancet Oncol.

[50] Kaufman PA, Awada A, Twelves C, Yelle L, Perez EA, Velikova G, Olivo MS, He Y, Dutcus CE, Cortes J (2015). Phase III open-label randomized study of eribulin mesylate versus capecitabine in patients with locally advanced or metastatic breast cancer previously treated with an anthracycline and a taxane. J Clin Oncol.

[51] Cortes J, Hudgens S, Twelves C, Perez EA, Awada A, Yelle L, McCutcheon S, Kaufman PA, Forsythe A, Velikova G (2015). Health-related quality of life in patients with locally advanced or metastatic breast cancer treated with eribulin mesylate or capecitabine in an open-label randomized phase 3 trial. Breast cancer research and treatment.

[52] Muller V, Fuxius S, Steffens CC, Lerchenmuller C, Luhn B, Vehling-Kaiser U, Hurst U, Hahn LJ, Soeling U, Wohlfarth T, Zaiss M (2014). Quality of life under capecitabine (Xeloda(R)) in patients with metastatic breast cancer: data from a german non-interventional surveillance study. Oncology research and treatment.

[53] Avallone A, Piccirillo MC, Delrio P, Pecori B, Di Gennaro E, Aloj L, Tatangelo F, D'Angelo V, Granata C, Cavalcanti E, Maurea N, Maiolino P, Bianco F (2014). Phase 1/2 study of valproic acid and short-course radiotherapy plus capecitabine as preoperative treatment in low-moderate risk rectal cancer-V-shoRT-R3 (Valproic acid--short Radiotherapy--rectum 3rd trial). BMC cancer.

[54] Bruzzese F, Di Gennaro E, Avallone A, Pepe S, Arra C, Caraglia M, Tagliaferri P, Budillon A (2006). Synergistic antitumor activity of epidermal growth factor receptor tyrosine kinase inhibitor gefitinib and IFN-alpha in head and neck cancer cells in vitro and in vivo. Clin Cancer Res.

[55] Bruzzese F, Pucci B, Milone MR, Ciardiello C, Franco R, Chianese MI, Rocco M, Di Gennaro E, Leone A, Luciano A, Arra C, Santini D, Caraglia M (2013). Panobinostat synergizes with zoledronic acid in prostate cancer and multiple myeloma models by increasing ROS and modulating mevalonate and p38-MAPK pathways. Cell death & disease.

[56] Burgenske DM, Monsma DJ, Dylewski D, Scott SB, Sayfie AD, Kim DG, Luchtefeld M, Martin KR, Stephenson P, Hostetter G, Dujovny N, MacKeigan JP (2014). Establishment of genetically diverse patient-derived xenografts of colorectal cancer. American journal of cancer research.

