# Supplementary material for: Valproic acid potentiates the anticancer activity of capecitabine in vitro and in vivo in breast cancer models via induction of thymidine phosphorylase expression
Source: Oncotarget. 2015 Dec 31;7(7):7715–31. doi: 10.18632/oncotarget.6802 (PMC4884949; doi:10.18632/oncotarget.6802)
Supplement: Supplementary file 1 [file oncotarget-07-7715-s001.pdf]

SUPPLEMENTARY FIGURES

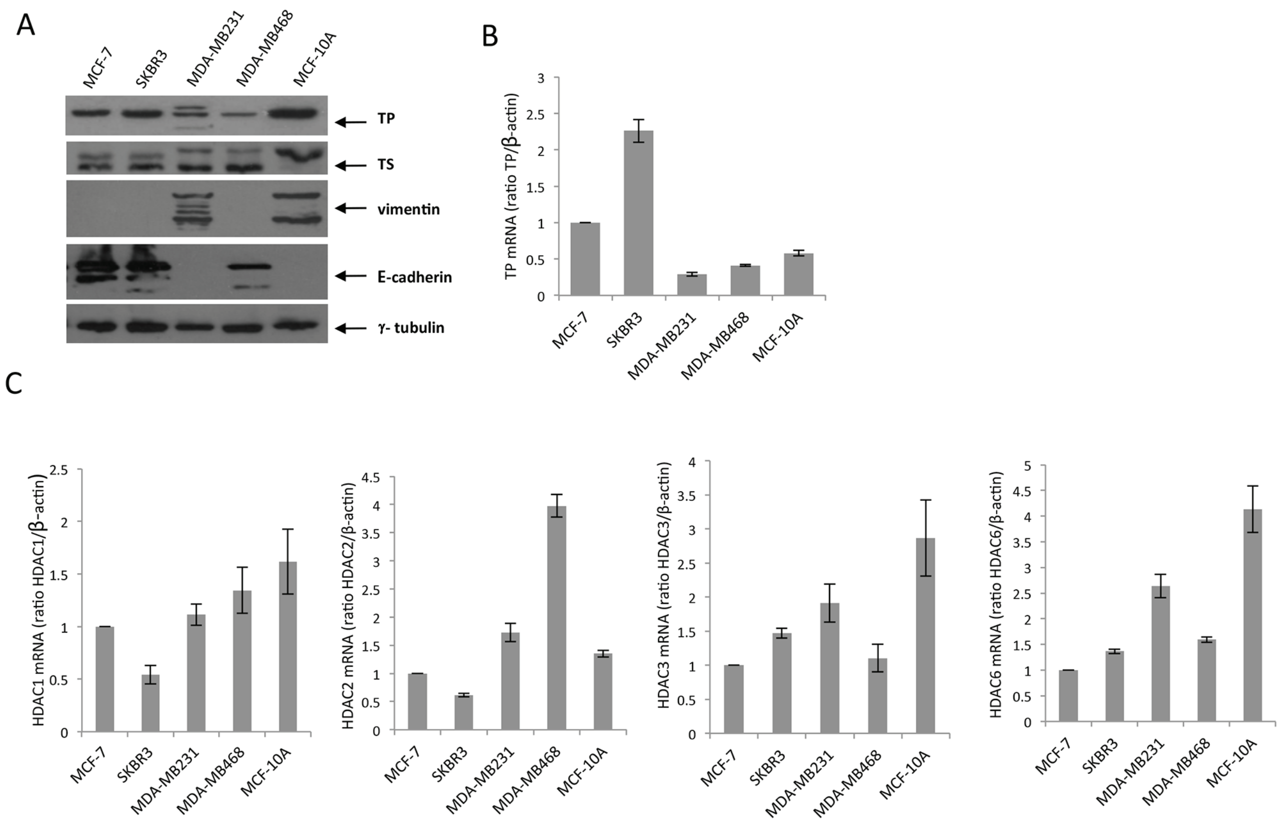

Supplementary Figure S1: TP, TS and HDACs basal expression in breast cancer cell lines. A. Basal expression of TP, TS, vimentin and E-cadherin proteins evaluated by western blot in MCF-7, SKBR3, MDA-MB231, MDA-MB468 and MCF-10A cells. γ-tubulin was used as protein loading control. **B.** Basal TP mRNA expression determined by qRealTime-PCR in MCF-7, SKBR3, MDA-MB231, MDA-MB468 and MCF-10A cells. **C.** Basal HDAC1, HDAC2, HDAC3 and HDAC6 mRNA expression determined by qRealTime-PCR in the same cell lines. β-actin was used as housekeeping control gene to normalize qRealTime-PCR reactions.

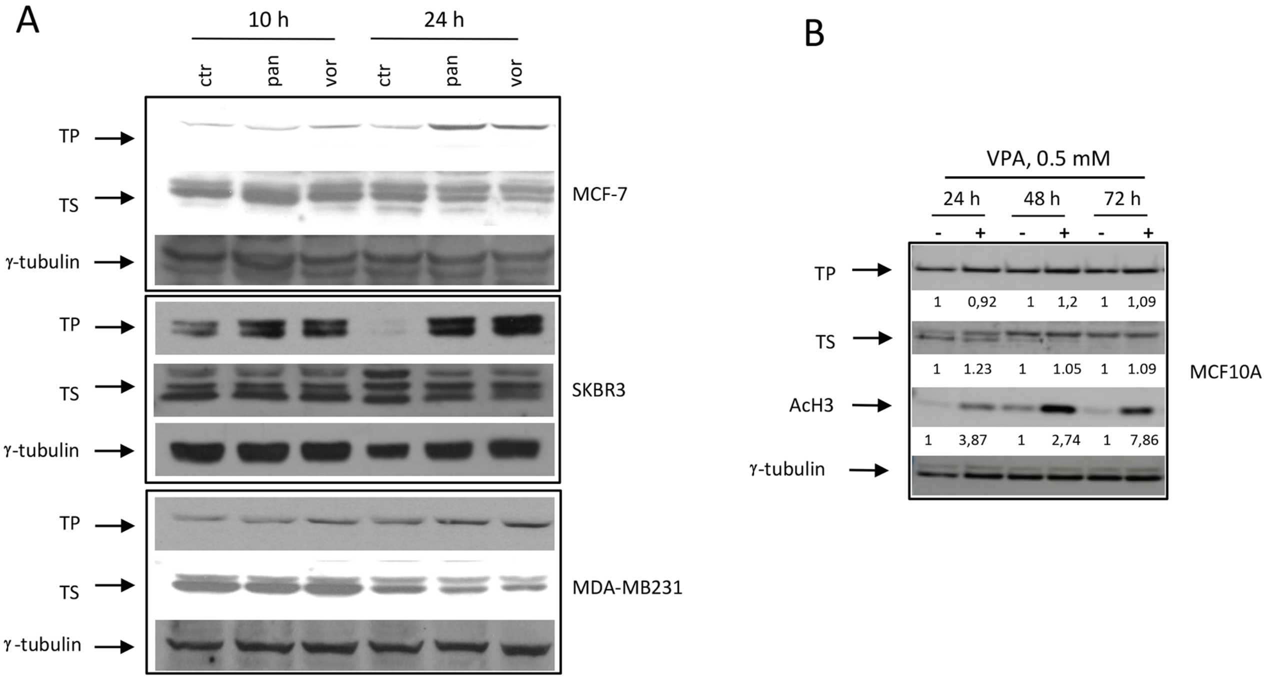

Supplementary Figure S2: HDACi modulates TS and TP protein expression in breast cancer cell lines in a time-dependent manner, but not in non-tumorigenic MCF-10A. A. TP and TS proteins evaluated by western blot in MCF-7, SKBR3 and MDA-MB231 cells untreated or treated for 10 or 24 hours with panobinostat or vorinostat IC_{50}^{72h}. **B.** TP, TS and acetyl-H3 proteins evaluated by western blot in non-tumorigenic MCF-10A cells untreated or treated with VPA 0.5 mM for 24, 48 and 72 hours, γ-Tubulin was used as protein loading control.

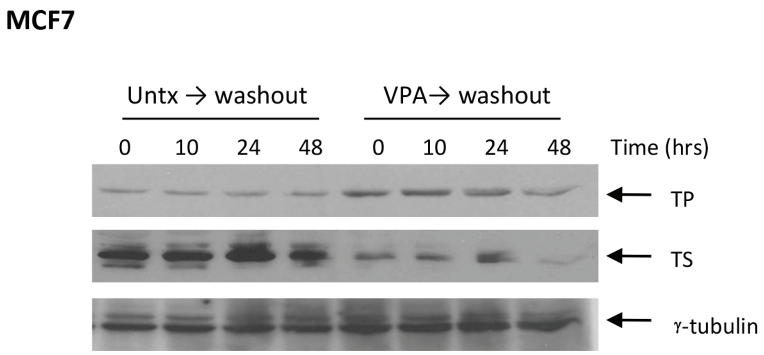

Supplementary Figure S3: VPA effect on TP and TS expression after time-course drug removal. TP and TS proteins determined by western blot in MCF-7 cells untreated or treated with VPA 2,5 mM for 24 hours (unit t = 0). Cells were then washed four times with PBS, placed in fresh media and collected at indicated times (t = 10, 24 and 48 hours).

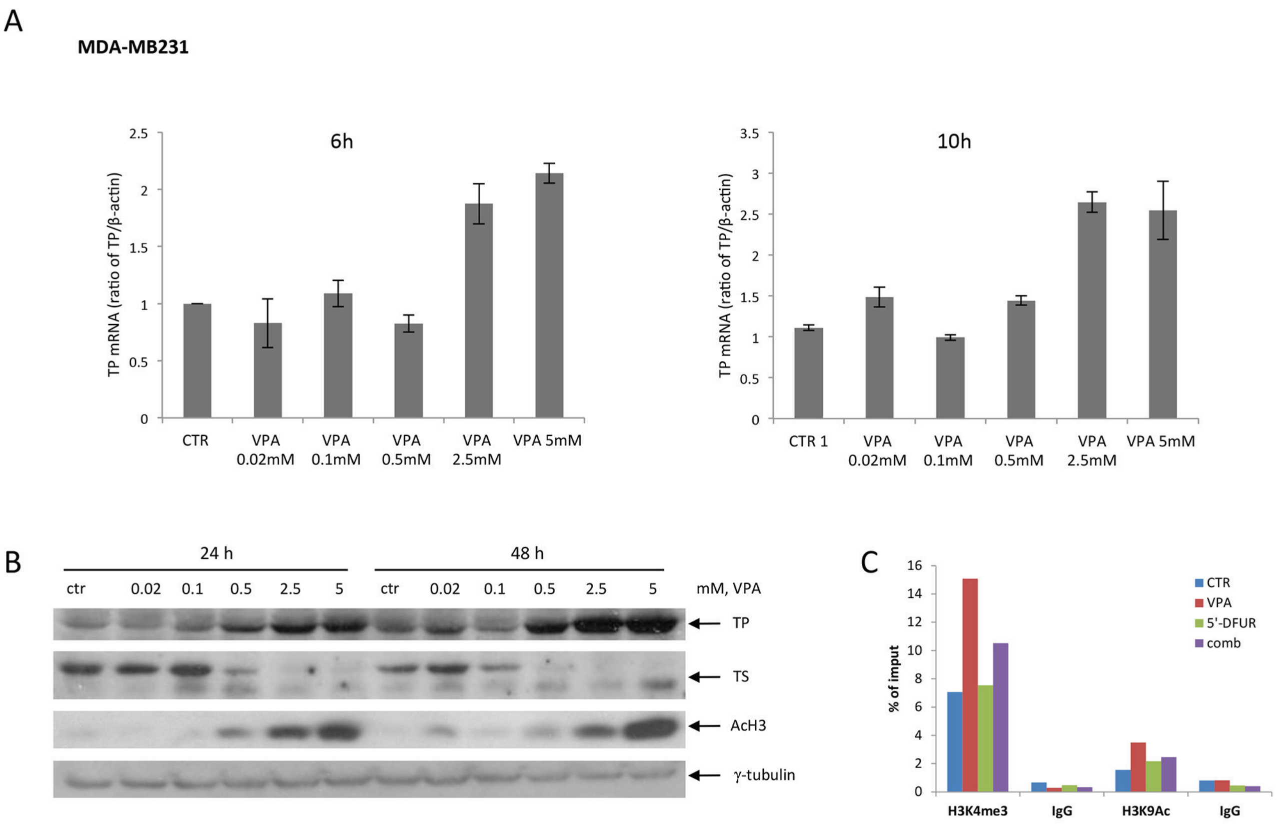

Supplementary Figure S4: HDACi VPA modulates TP and TS expression in MDA-MB231 cells. A. TP and TS mRNA expression evaluated by qRealTime-PCR on MCF-7 cells treated or untreated with VPA at the indicated concentrations for 6 and 10 hours. **B.** TP, TS and acetyl-H3 proteins evaluated in MCF-7 cells untreated or treated for 24 and 48 hours with VPA at the indicated concentrations. γ-Tubulin was used as protein loading control. **C.** ChiP results were used to measure the levels of two active histone marks at TP promoter. Acetylation of Histone 3 lysine 9 (H3K9Ac) and trimethylation of Histone 3 lysine 4, (H3-K4me3), at the promoter of TP gene in MDA-MB231 cells untreated or treated for 24 hours with 2 mM VPA and/or 1.5 µM 5′-DFUR.

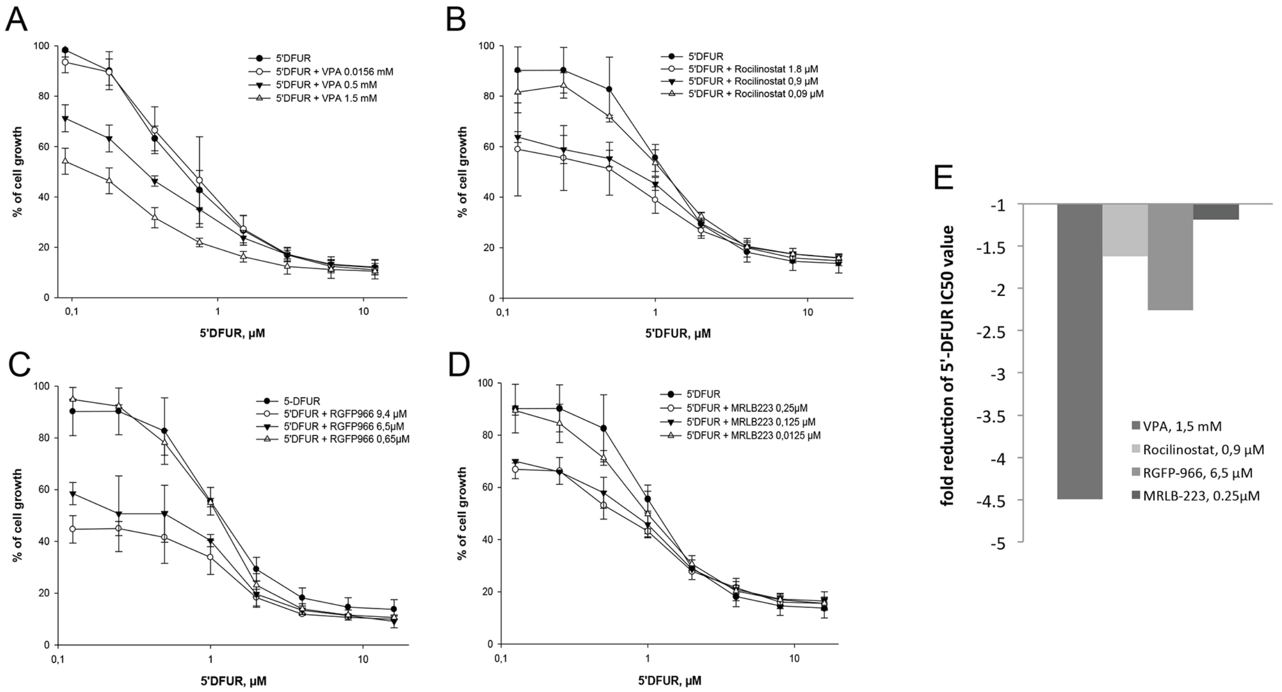

Supplementary Figure S5: Antiproliferative effects of 5′-DFUR in combination with low doses of VPA, rocilinostat, RGFP-966 or MRLB-223 in MCF-7 cells. Cells were incubated with increased doses of 5′-DFUR and/or fixed doses of VPA **A.** rocilinostat **B.** RGFP-966 **C.** or MRLB-223 **D.** for 96 hours. Cell viability, assessed by the SRB method, is expressed as % of control for each time point. Values are the mean of at least two experiments performed in quadruplicates. **E.** Fold reduction of 5′-DFUR IC_{50} value was calculated as ratio between 5′-DFUR IC_{50} value as single agent and the IC_{50} of 5′-DFUR in combination with HDACi at IC_{30}.

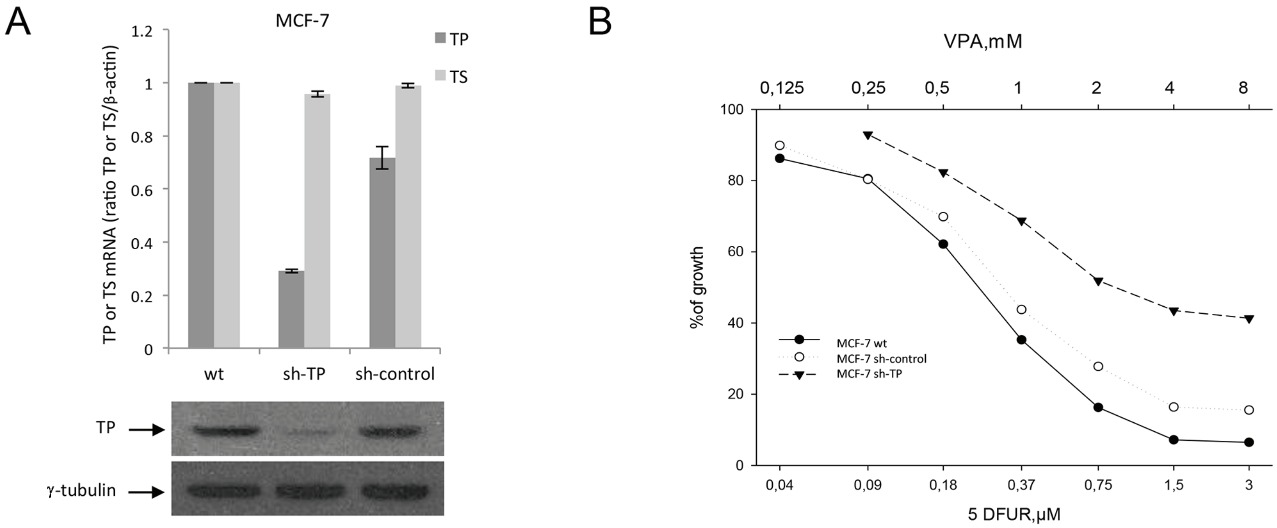

Supplementary Figure S6: TP protein expression plays a critical role in synergistic antiproliferative effect induced by VPA/5′-DFUR combination. A. TP and TS mRNA expression evaluated by qRealTime-PCR and TP protein expression evaluated by western blot on MCF-7 cells untrasfected (wt) or transfected with TP-specific shRNA (sh-TP) or control shRNA (sh-control). β-actin was used as housekeeping control gene to normalize qRealTime-PCR reactions and γ-Tubulin was used as protein loading control. **B.** Antiproliferative effect of VPA/5′-DFUR combination on MCF-7 wt, MCF-7 sh-control and MCF-7 sh-TP cells. Cell viability, assessed by the SRB method, is expressed as % of control for each time point. Values are the mean of an experiment performed in quadruplicates.

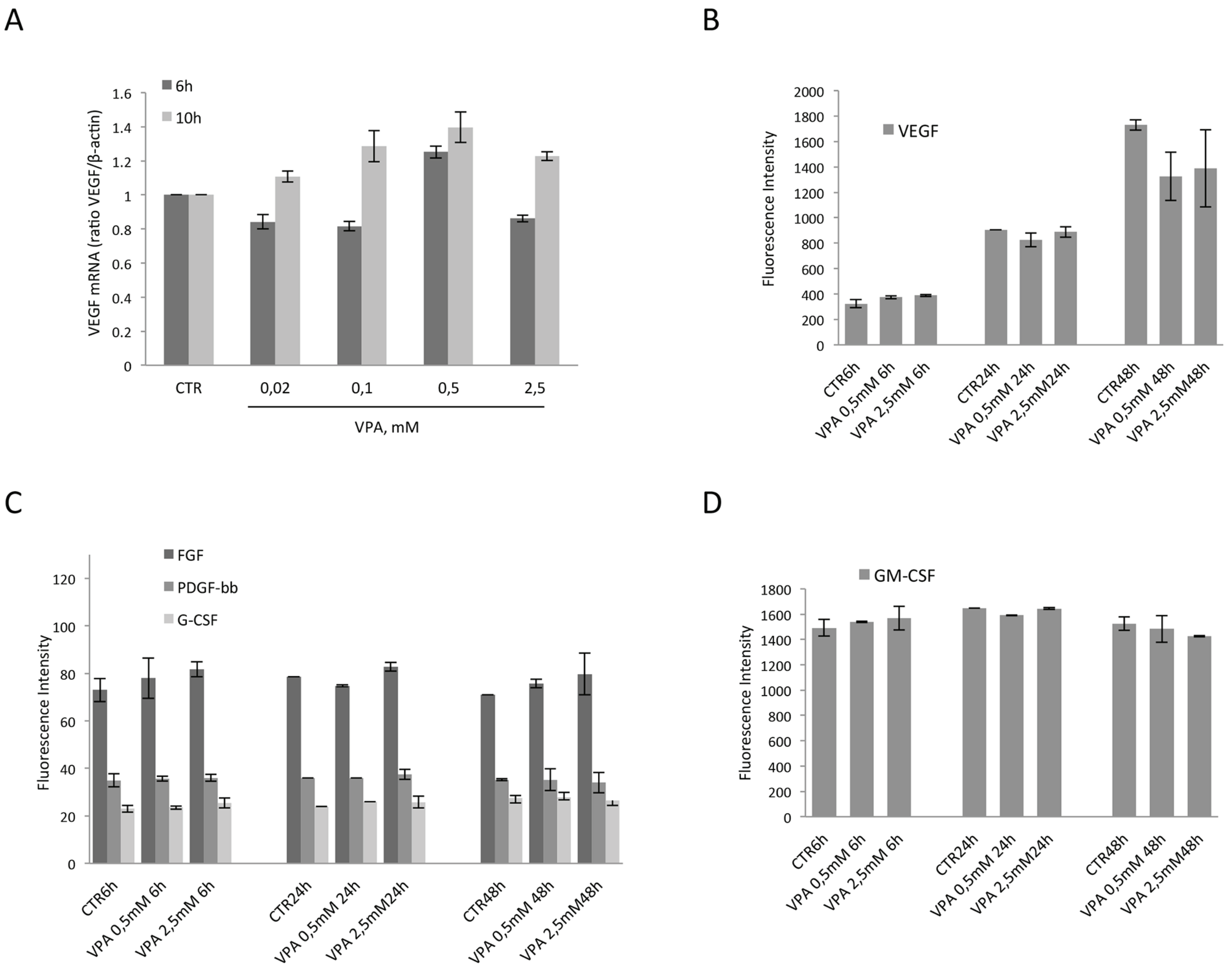

Supplementary Figure S7: Induction of TP mediated by VPA treatment did not induce angiogenic factors. A. VEGF mRNA expression evaluated by q-RT-PCR on MCF-7cells untreated or treated with VPA at indicated concentrations for 6 and 10 hours. **B.C.** and **D.** VEGF (B), GM-CSF, FGF, PDGF-bb (C) and GM-CSF (D) analyzed in cell supernatant using Bio-Plex™ Pro Human Cytokine 27-Plex assay on supernatant of MCF-7 celle untreated or treated with VPA 0.5 mM for 6, 24 or 48 hours.

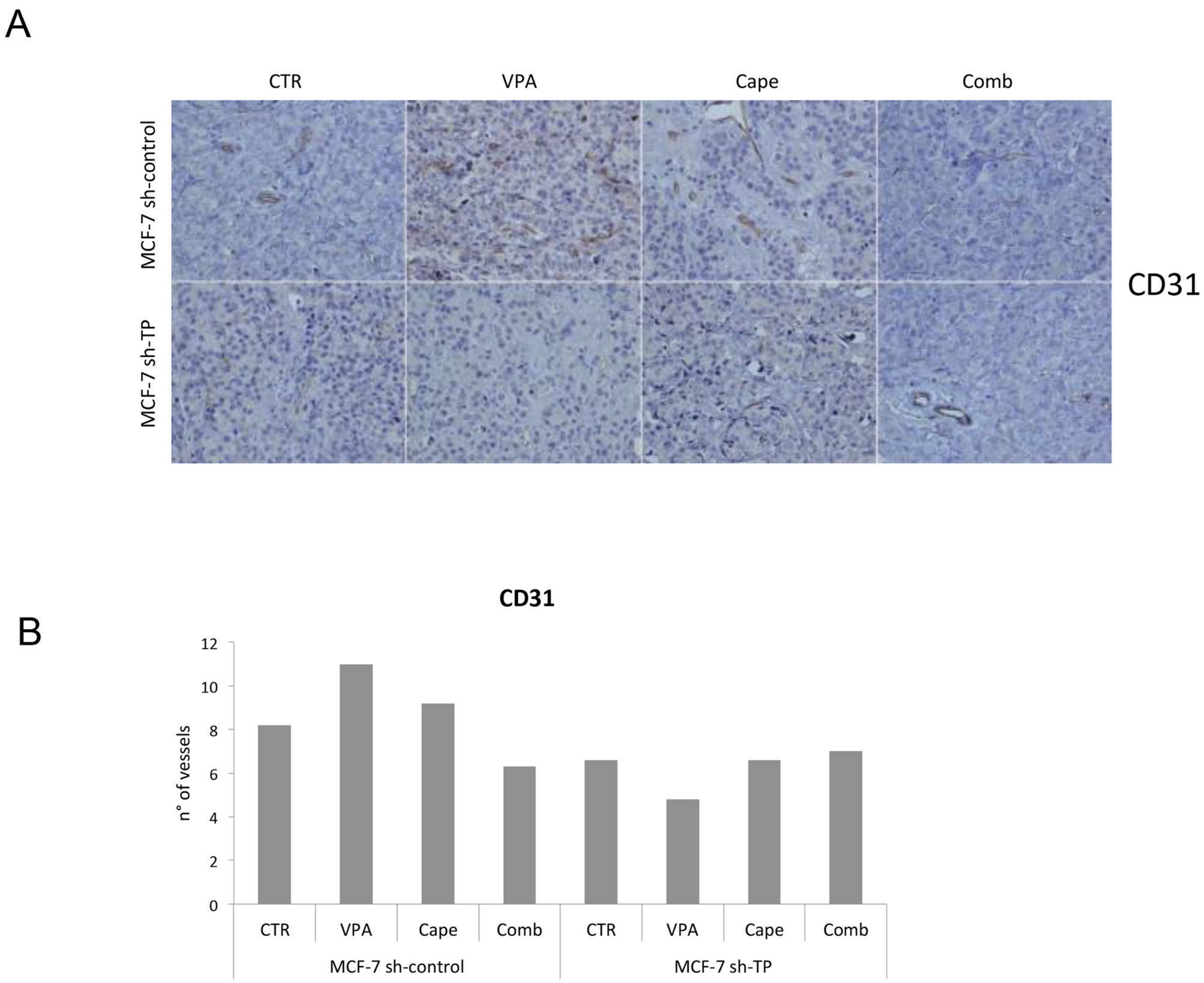

Supplementary Figure S8: *In vivo* VPA/capecitabine treatment did not induce angiogenesis. A. Paraffin-embedded tissues were generated for each group for hematoxylin and eosin stain (H&E) and immunohistochemistry analysis for CD31 as described in the Materials and Methods. Images were captured with a 20x or 40x objective on a light microscope. **B.** Tumour sections stained for CD31 were scored semi-quantitatively for the number vessels.
